# Supplementary material for: Sustainable and scalable synthesis of polysubstituted bis-1,2,4-triazoles, bis-2-iminothiazolines and bis-thiobarbiturates using bis-N,N-disubstituted thioureas as versatile substrate
Source: R Soc Open Sci. 2019 Jun 19;6(6):181963. doi: 10.1098/rsos.181963 (PMC6599792; doi:10.1098/rsos.181963)
Supplement: Supplementary information [file rsos181963supp1.pdf]

# Electronic Supporting Information

## Sustainable and scalable synthesis of polysubstituted bis-1,2,4-triazoles, bis-2-iminothiazolines and bis-thiobarbiturates utilizing bis-*N*,*N*-disubstituted thioureas as versatile substrate

Wael Abdelgayed Ahmed Arafa*[a, b] and Hamada Mohamed Ibrahim[b]

[a]Chemistry Department, College of Science, Jouf University, P.O. Box 2014, Sakaka, Aljouf, Kingdom of Saudi Arabia

[b]Chemistry Department, Faculty of Science, Fayoum University P.O. Box 63514, Fayoum City, Egypt

E-mail:waa00@fayoum.edu.eg


| No. | Content | Page No. |
|---|---|---|
| 1. | Experimental details for **3a-d** synthesis | S2-S3 |
| 2. | $^{1}$H NMR of **3a** | S4 |
| 3. | $^{1}$H NMR of **3b** | S5 |
| 4. | $^{13}$C NMR of **3b** | S6 |
| 5. | $^{1}$H NMR of **3c** | S7 |
| 6. | $^{13}$C NMR of **3c** | S8 |
| 7. | $^{1}$H NMR of **3d** | S9 |
| 8. | $^{13}$C NMR of **3d** | S10 |
| 9. | $^{1}$H NMR of **4a** | S11 |
| 10. | $^{1}$H NMR of **4b** | S12 |
| 11. | $^{1}$H NMR of 4**c+5a** | S13 |
| 12. | $^{1}$H NMR of **4d + 5b** | S14 |
| 13. | $^{1}$H NMR of **6** | S15 |
| 14. | $^{1}$H NMR of **7a** | S16 |

| | | |
|---|---|---|
| 15. | $^{1}$H NMR of **7b** | S17 |
| 16. | $^{1}$H NMR of **8a + 9a** | S18 |
| 17. | $^{1}$H NMR of **8b+9b** | S19 |
| 18. | $^{1}$H NMR of **10a** | S20 |
| 19. | $^{1}$H NMR of **10b** | S21 |
| 20. | $^{1}$H NMR of **10c** | S22 |
| 21. | $^{1}$H NMR of **10d** | S23 |
| 22. | $^{1}$H NMR of **11a** | S24 |
| 23. | $^{1}$H NMR of **11b** | S25 |
| 24. | $^{1}$H NMR of **11c** | S26 |
| 25. | $^{1}$H NMR of **11d** | S27 |
| 26. | $^{1}$H NMR of **13a** | S28 |
| 27. | $^{1}$H NMR of **13b** | S29 |
| 28. | $^{1}$H NMR of **13c** | S30 |
| 29. | $^{1}$H NMR of **13d** | S31 |
| 30. | Green Metrics Calculations and References | S32 |


**General procedure for the synthesis of bis-thioureas (3a–d).**

Isothiocyanates (**1a**, **b**, 0.5 mmol) and diamines (**2a**, **b**, 1.0 mmol) were mixed in a 25 mL round-bottomed flask. The reaction mixture was sonicated in the water bath (23 $^{o}$C) of an ultrasonic cleaner, for 5 min as indicated in Table 1. After completion of the reaction (the reaction was monitored by TLC, silica gel; DCM:petroleum ether = 5:2 V/V), the reaction mixture was triturated with 2 mL chilled EtOH, and the precipitate was filtered, affording the crude product, which was purified by recrystallization from EtOH (95–97%).

**1,1'-((1*R*,4*R*)-Cyclohexane-1,4-diyl)bis(3-benzylthiourea)** (**3a**), colorless solid, m.p. 229–231 $^{o}$C; $^{1}$H NMR (400 MHz, DMSO-$d_6$) δ (ppm): 7.65 (b, 2H, 2NH), 7.35-7.21 (m, 12H, 2NH + Ar-H), 4.66 (d, $J$ = 5.1, 4H, $2CH_2$), 3.92 (b, 2H,

cyclohexyl-H), 1.95–1.93 (m, 4H, cyclohexyl-H), 1.27–1.21 (m, 4H, cyclohexyl-H); $^{13}$C NMR (100 MHz, DMSO-*d*$_6$) δ (ppm): 181.4, 139.2, 127.9, 127.4, 126.4, 51.5, 47.0, 30.5; IR (KBr, cm$^{-1}$): 3265, 3221 (NH), 1573 (C=C), 1262 (C=S); MS *m/z* (%): 412 (0.4), 321 (24.2), 248 (12.8), 165 (57.4), 91 (100); Anal. Calcd for $C_{22}H_{28}N_4S_2$: C, 64.04; H, 6.84; N, 13.58%; Found: C, 64.09; H, 6.79; N, 13.62%.

**1,1'-((1*R*,4*R*)-Cyclohexane-1,4-diyl)bis(3-allylthiourea)** (**3b**), pale yellow solid, m.p. 330–332 °C; $^{1}$H NMR (400 MHz, DMSO-*d*$_6$) δ (ppm): 7.33 (b, 2H, 2NH), 7.27–7.25 (d, 2H, 2NH), 5.90–5.77 (m, 2H, $CH_2$=C*H*), 5.16–5.04 (m, 4H, C*H*$_2$=CH), 4.03 (b, 4H, C*H*$_2$–N), 3.91 (b, 2H, cyclohexyl-H), 1.92–1.90 (m, 4H, cyclohexyl-H), 1.25–1.19 (m, 4H, cyclohexyl-H); $^{13}$C NMR (100 MHz, DMSO-*d*$_6$) δ (ppm): 181.1, 135.1, 115.3, 51.4, 45.7, 30.8; IR (KBr, cm$^{-1}$): 3237, 3211 (NH), 1569 (C=C), 1260 (C=S); MS *m/z* (%): 312 (0.1), 300 (0.2), 256 (40.4), 198 (26.9), 117 (33.4), 96 (49.7), 58 (100); Anal. Calcd for $C_{14}H_{24}N_4S_2$: C, 53.81; H, 7.74; N, 17.93%; Found: C, 53.77; H, 7.80; N, 17.85%.

**1,1'-(1,4-Phenylenebis(methylene))bis(3-benzylthiourea)** (**3c**), light yellow solid, m.p. 166–167 °C; $^{1}$H NMR (400 MHz, DMSO-*d*$_6$) δ (ppm): 7.89 (b, 4H, 4NH), 7.35–7.23 (m, 14H, Ar-H), 4.66 (b, 8H, $4CH_2$); $^{13}$C NMR (100 MHz, DMSO-*d*$_6$) δ (ppm): 181.5, 139.2, 137.8, 128.2, 127.3, 127.2, 126.8, 47.0, 46.8; IR (KBr, cm$^{-1}$): 3271, 3232 (NH), 1562 (C=C), 1250 (C=S); MS *m/z* (%): 434 (0.6), 343 (21.6), 252 (58.3), 104 (23.5), 91 (100); Anal. Calcd for $C_{24}H_{26}N_4S_2$: C, 66.33; H, 6.03; N, 12.89%; Found: C, 66.42; H, 5.95; N, 12.79%.

**1,1'-(1,4-Phenylenebis(methylene))bis(3-allylthiourea)** (**3d**), yellow solid, m.p. 176–178 °C; $^{1}$H NMR (400 MHz, DMSO-*d*$_6$) δ (ppm): 7.80 (b, 2H, 2NH), 7.55 (b, 2H, 2NH), 7.23 (s, 4H, Ar-H), 5.89–5.80 (m, 2H, $CH_2$=C*H*), 5.17–5.05 (m, 4H, C*H*$_2$=CH), 4.63 (d, $J$ = 5.0 Hz, 4H, $2CH_2$), 4.06 (b, 4H, C*H*$_2$–N); $^{13}$C NMR (100

MHz, DMSO-$d_6$) δ (ppm): 181.2, 137.8, 135.0, 127.1, 115.3, 46.7, 45.8; IR (KBr, $cm^{-1}$): 3267, 3224 (NH), 1562 (C=C), 1259 (C=S); MS *m/z* (%): 334(0.3), 279 (1.6), 262 (31.4), 99 (43.6), 91 (88.7), 56 (100); Anal. Calcd for $C_{16}H_{22}N_4S_2$: C, 57.45; H, 6.63; N, 16.75%; Found: C, 57.51; H, 6.57; N, 16.83%.

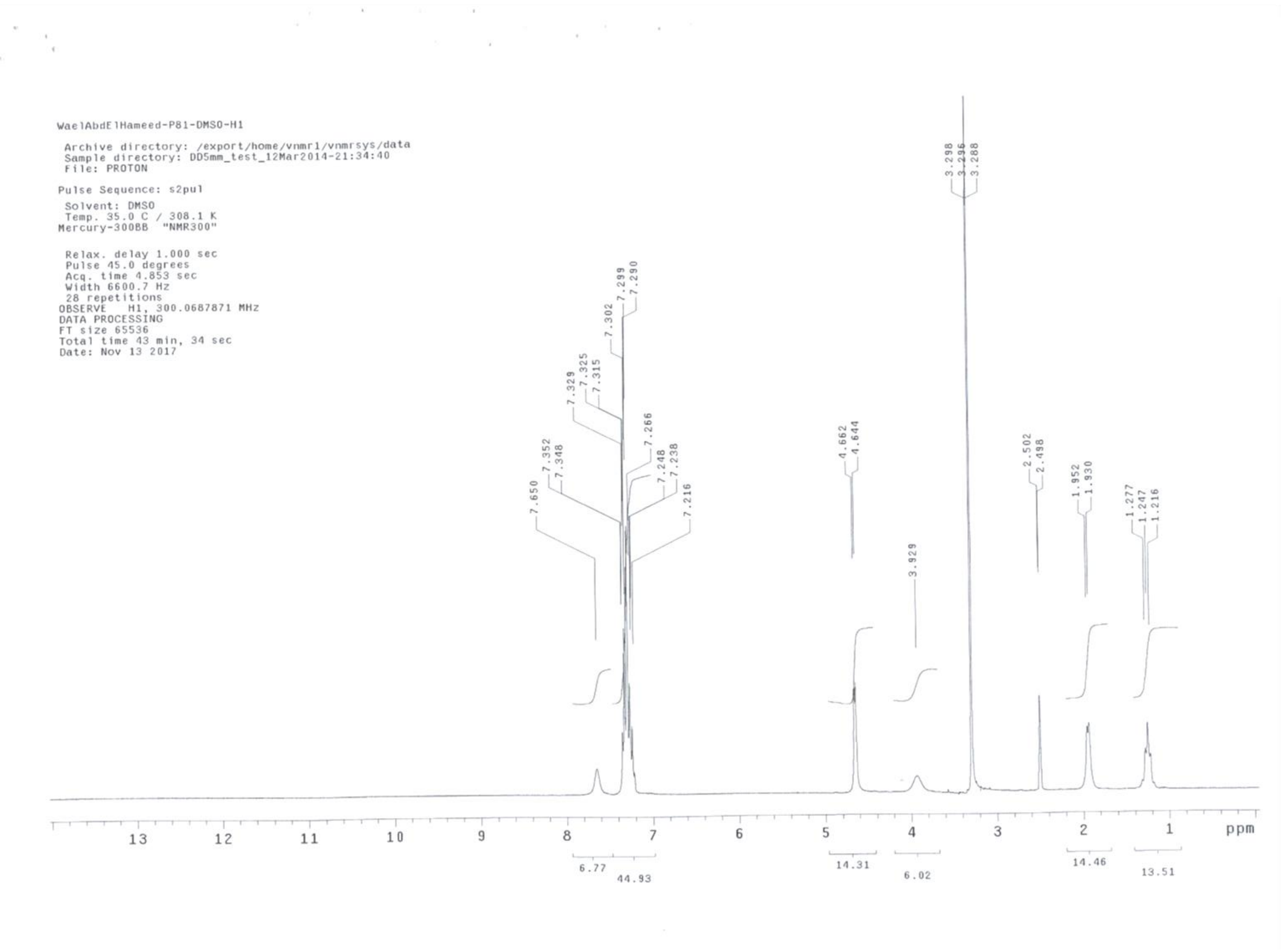


$^1$H NMR of **3a**

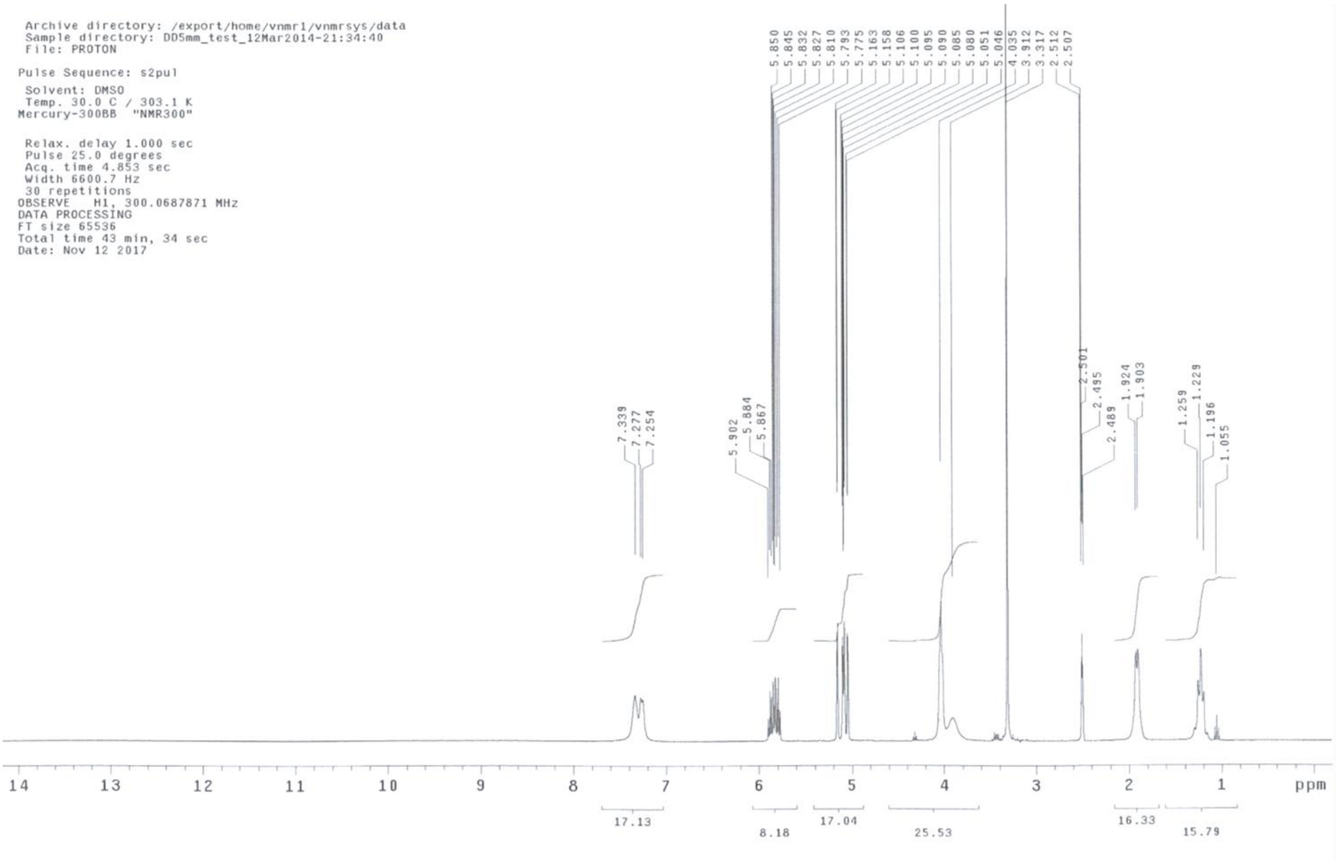
Archive directory: /export/home/vnmr1/vnmrsys/data
Sample directory: DD5mm_test_12Mar2014-21:34:40
File: PROTON
Pulse Sequence: s2pul
Solvent: DMSO
Temp. 30.0 C / 303.1 K
Mercury-300BB "NMR300"
Relax. delay 1.000 sec
Pulse 25.0 degrees
Acq. time 4.853 sec
Width 6600.7 Hz
30 repetitions
OBSERVE H1, 300.0687871 MHz
DATA PROCESSING
FT size 65536
Total time 43 min, 34 sec
Date: Nov 12 2017
ppm
17.13
8.18
17.04
25.53
16.33
15.79

$^1$H NMR of **3b**

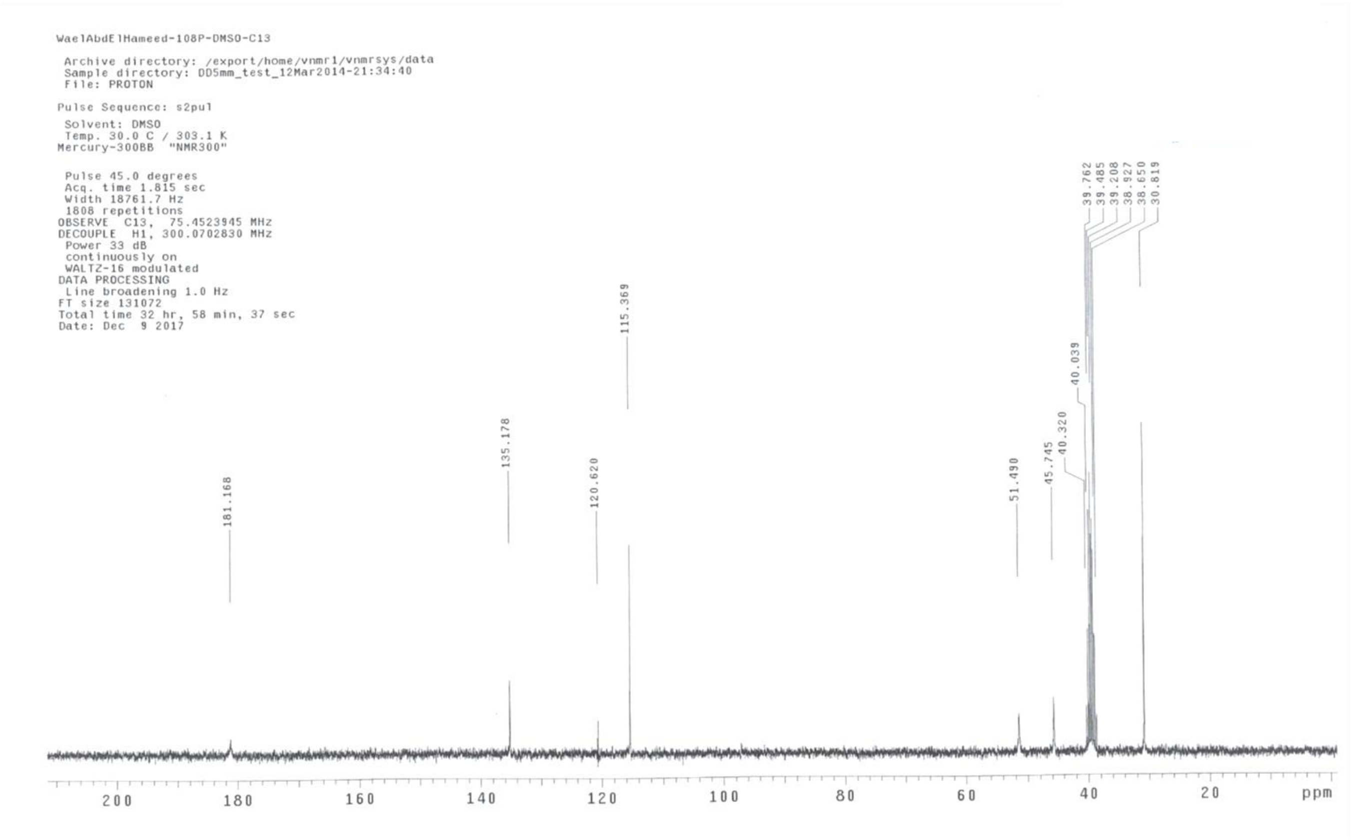
WaelAbdElHameed-108P-DMSO-C13
Archive directory: /export/home/vnmr1/vnmrsys/data
Sample directory: DD5mm_test_12Mar2014-21:34:40
File: PROTON
Pulse Sequence: s2pul
Solvent: DMSO
Temp. 30.0 C / 303.1 K
Mercury-300BB "NMR300"
Pulse 45.0 degrees
Acq. time 1.815 sec
Width 18761.7 Hz
1808 repetitions
OBSERVE C13, 75.4523945 MHz
DECOUPLE H1, 300.0702830 MHz
Power 33 dB
continuously on
WALTZ-16 modulated
DATA PROCESSING
Line broadening 1.0 Hz
FT size 131072
Total time 32 hr, 58 min, 37 sec
Date: Dec 9 2017
181.168
135.178
120.620
115.369
51.490
45.745
40.320
40.039
39.762
39.485
39.208
38.927
38.650
30.819
200
180
160
140
120
100
80
60
40
20
ppm

$^{13}$C NMR of **3b**

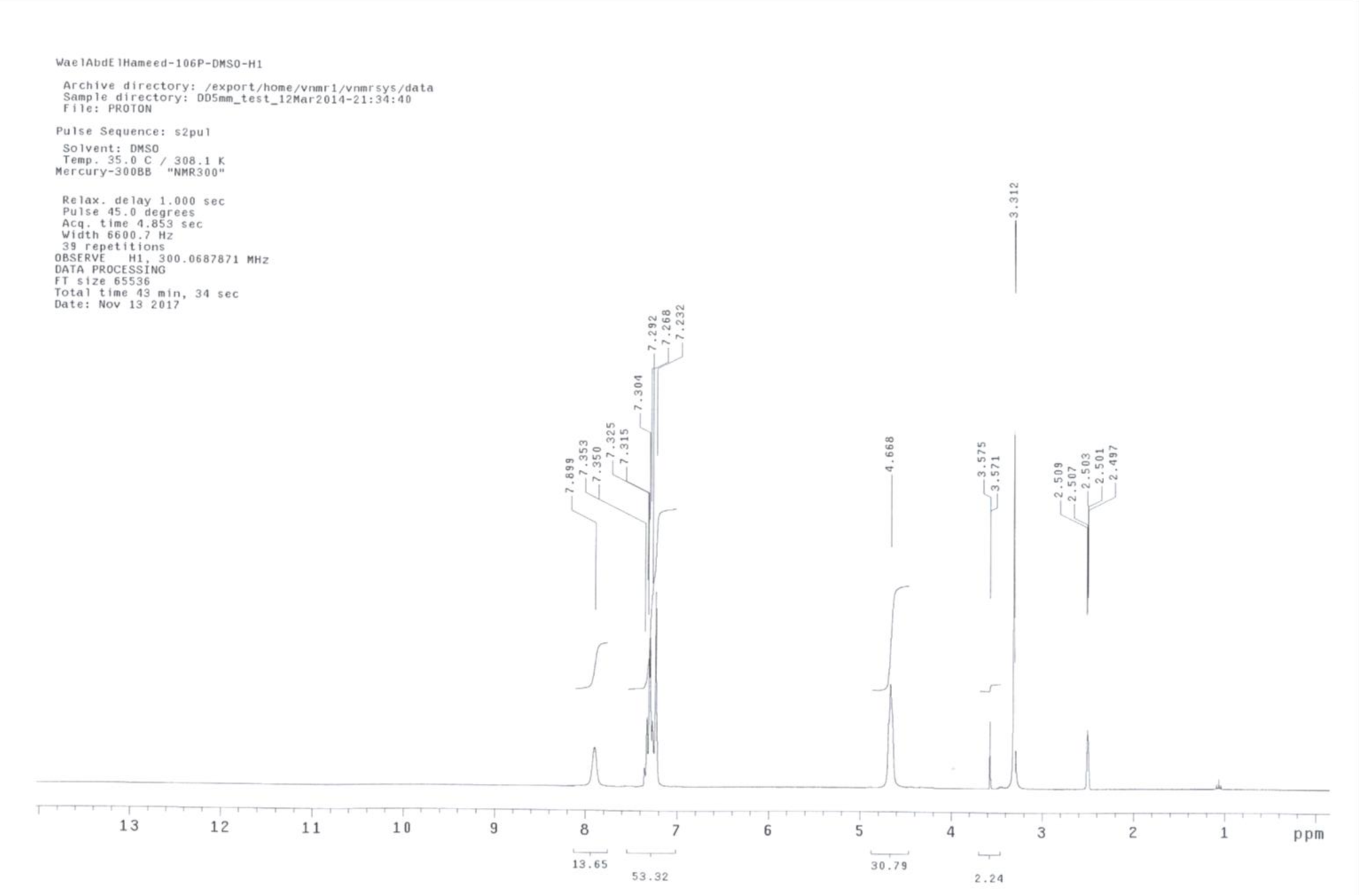
WaelAbdElHameed-106P-DMSO-H1
Archive directory: /export/home/vnmr1/vnmrsys/data
Sample directory: DD5mm_test_12Mar2014-21:34:40
File: PROTON
Pulse Sequence: s2pul
Solvent: DMSO
Temp. 35.0 C / 308.1 K
Mercury-300BB "NMR300"
Relax. delay 1.000 sec
Pulse 45.0 degrees
Acq. time 4.853 sec
Width 6600.7 Hz
39 repetitions
OBSERVE H1, 300.0687871 MHz
DATA PROCESSING
FT size 65536
Total time 43 min, 34 sec
Date: Nov 13 2017
7.899
7.353
7.350
7.325
7.315
7.304
7.292
7.268
7.232
4.668
3.575
3.571
3.312
2.509
2.507
2.503
2.501
2.497
13
12
11
10
9
8
7
6
5
4
3
2
1
ppm
13.65
53.32
30.79
2.24

$^{1}$H NMR of **3c**

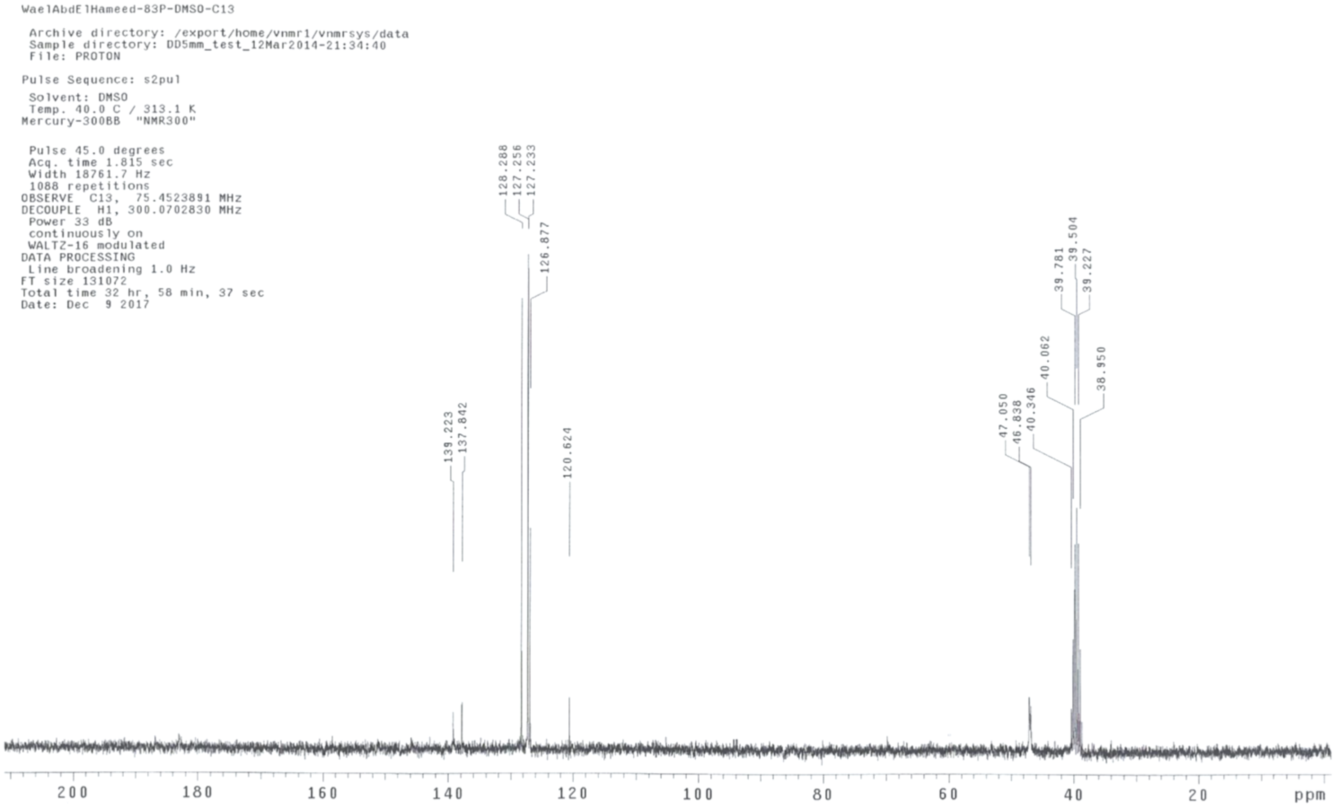
WaelAbdElHameed-83P-DMSO-C13
Archive directory: /export/home/vnmr1/vnmrsys/data
Sample directory: DD5mm_test_12Mar2014-21:34:40
File: PROTON
Pulse Sequence: s2pul
Solvent: DMSO
Temp. 40.0 C / 313.1 K
Mercury-300BB "NMR300"
Pulse 45.0 degrees
Acq. time 1.815 sec
Width 18761.7 Hz
1088 repetitions
OBSERVE C13, 75.4523891 MHz
DECOUPLE H1, 300.0702830 MHz
Power 33 dB
continuously on
WALTZ-16 modulated
DATA PROCESSING
Line broadening 1.0 Hz
FT size 131072
Total time 32 hr, 58 min, 37 sec
Date: Dec 9 2017
139.223
137.842
128.288
127.256
127.233
126.877
120.624
47.050
46.838
40.346
40.062
39.781
39.504
39.227
38.950
200
180
160
140
120
100
80
60
40
20
ppm

$^{13}$C NMR of **3c**

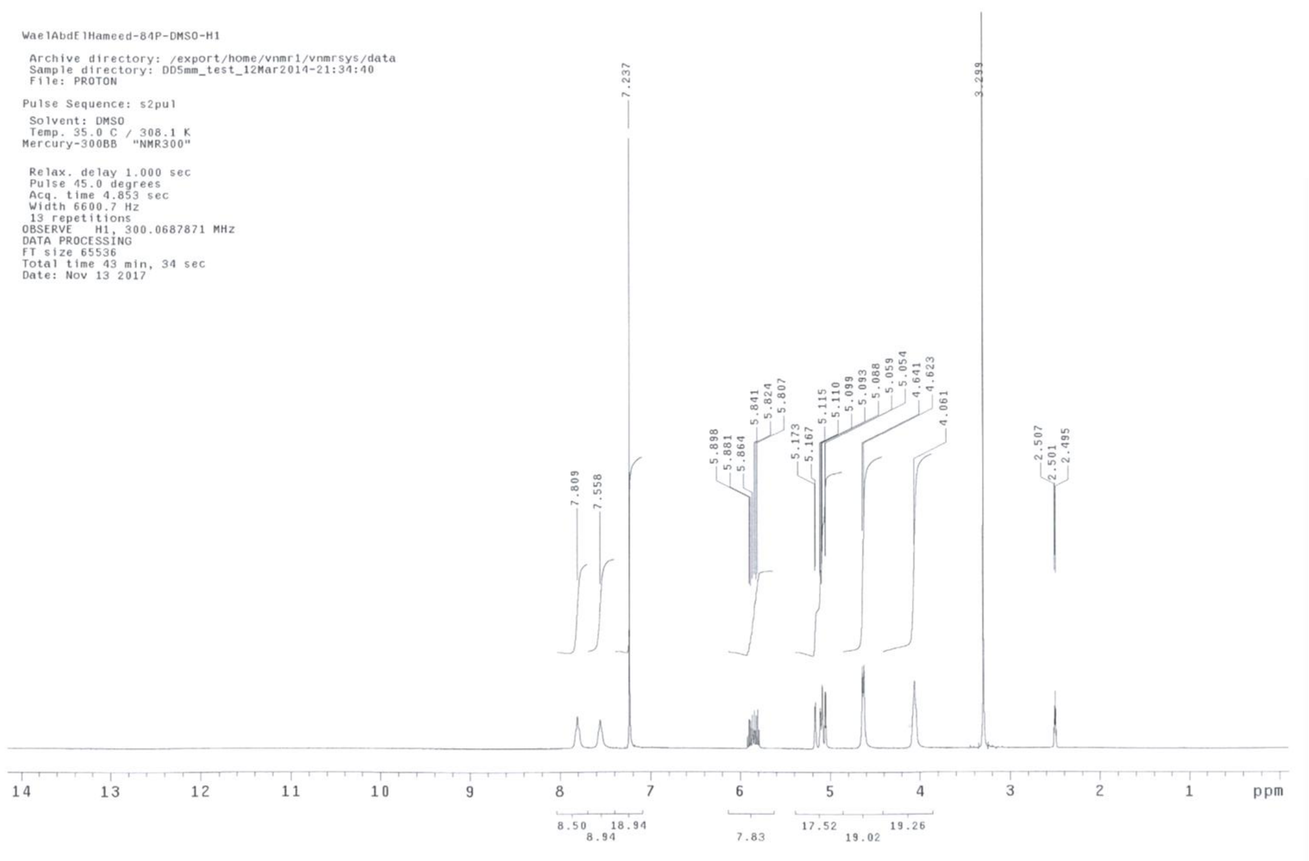
WaelAbdElHameed-84P-DMSO-H1
Archive directory: /export/home/vnmr1/vnmrsys/data
Sample directory: DD5mm_test_12Mar2014-21:34:40
File: PROTON
Pulse Sequence: s2pul
Solvent: DMSO
Temp. 35.0 C / 308.1 K
Mercury-300BB "NMR300"
Relax. delay 1.000 sec
Pulse 45.0 degrees
Acq. time 4.853 sec
Width 6600.7 Hz
13 repetitions
OBSERVE H1, 300.0687871 MHz
DATA PROCESSING
FT size 65536
Total time 43 min, 34 sec
Date: Nov 13 2017
ppm

$^{1}$H NMR of **3d**

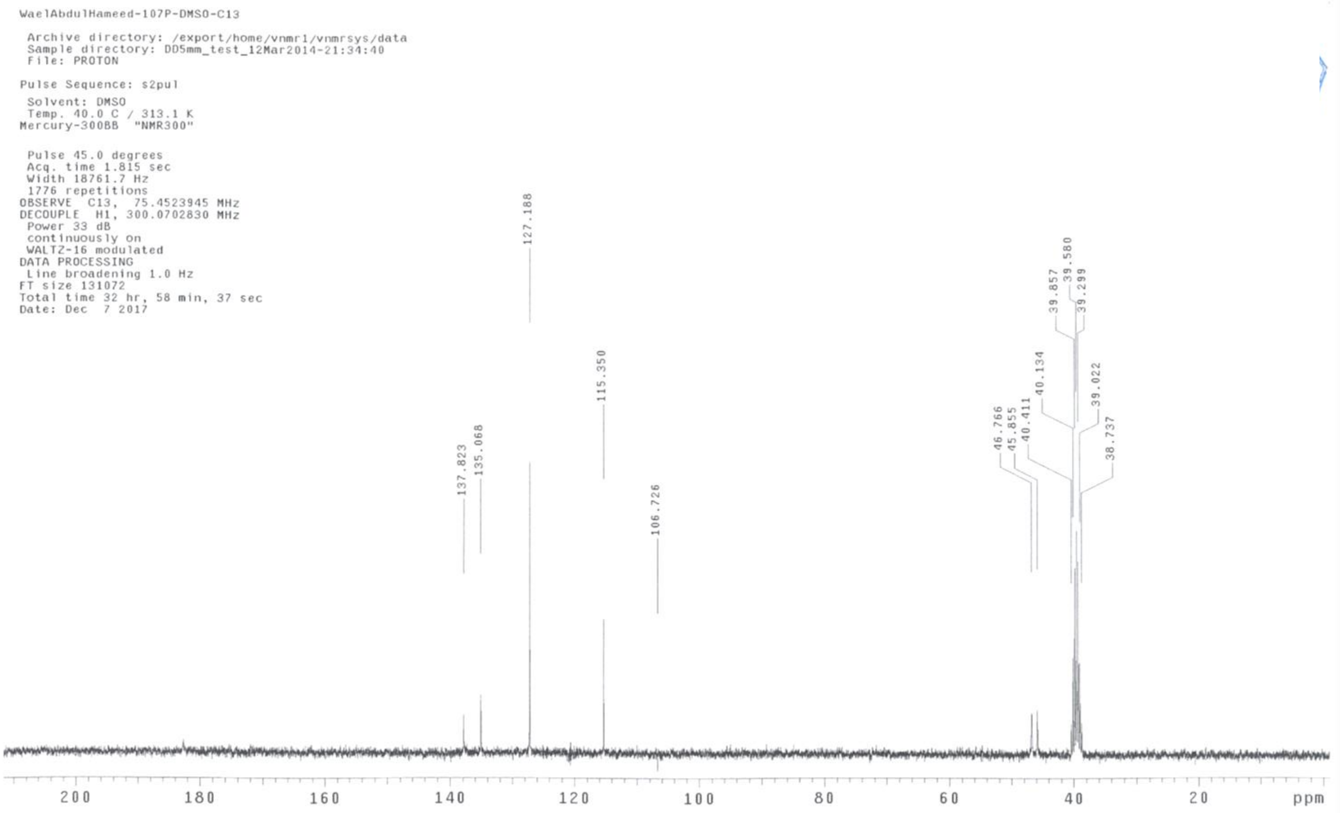
WaelAbdulHameed-107P-DMSO-C13
Archive directory: /export/home/vnmr1/vnmrsys/data
Sample directory: DD5mm_test_12Mar2014-21:34:40
File: PROTON
Pulse Sequence: s2pul
Solvent: DMSO
Temp. 40.0 C / 313.1 K
Mercury-300BB "NMR300"
Pulse 45.0 degrees
Acq. time 1.815 sec
Width 18761.7 Hz
1776 repetitions
OBSERVE C13, 75.4523945 MHz
DECOUPLE H1, 300.0702830 MHz
Power 33 dB
continuously on
WALTZ-16 modulated
DATA PROCESSING
Line broadening 1.0 Hz
FT size 131072
Total time 32 hr, 58 min, 37 sec
Date: Dec 7 2017
137.823
135.068
127.188
115.350
106.726
46.766
45.855
40.411
40.134
39.857
39.580
39.299
39.022
38.737
200
180
160
140
120
100
80
60
40
20
ppm

$^{13}$C NMR of **3d**

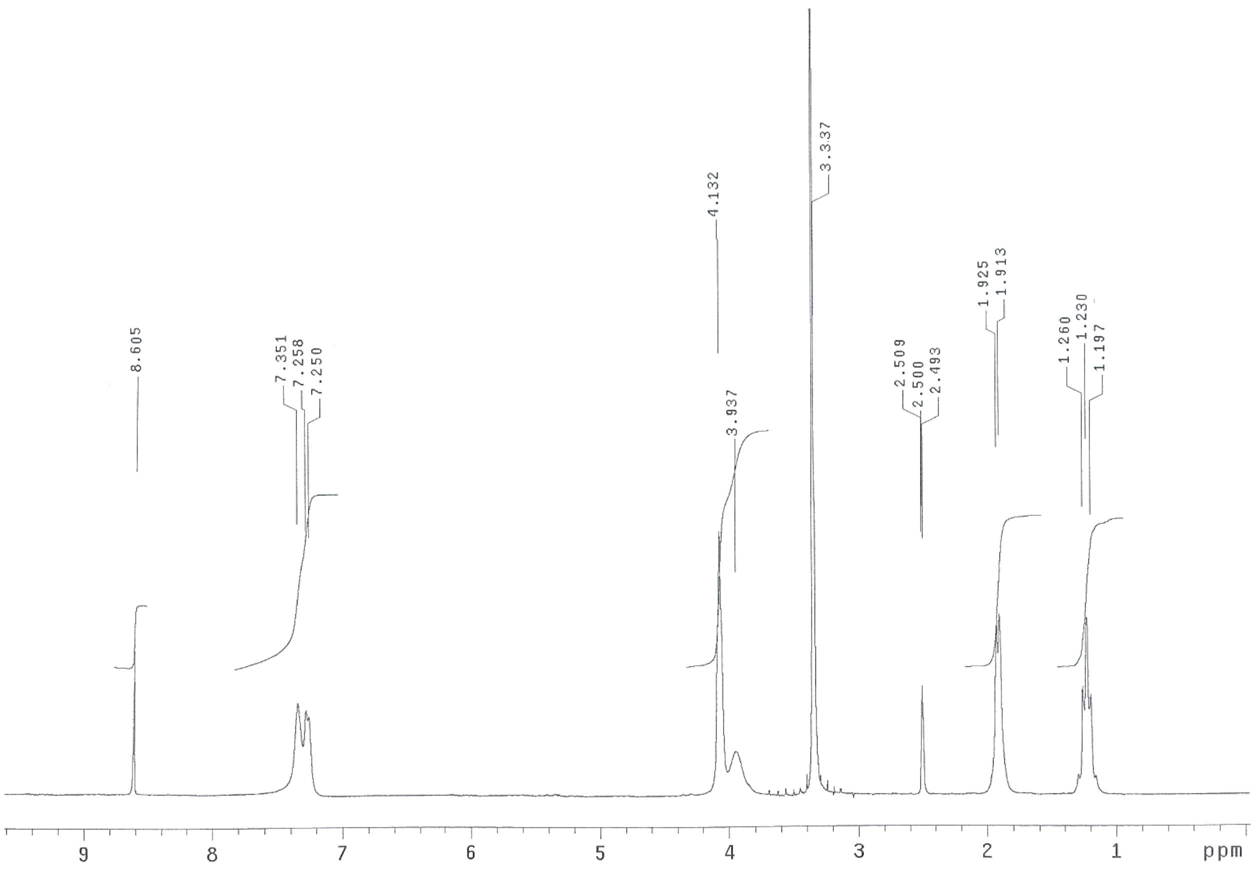
8.605
7.351
7.258
7.250
4.132
3.937
3.337
2.509
2.500
2.493
1.925
1.913
1.260
1.230
1.197
9
8
7
6
5
4
3
2
1
ppm

$^1$H NMR of **4a**

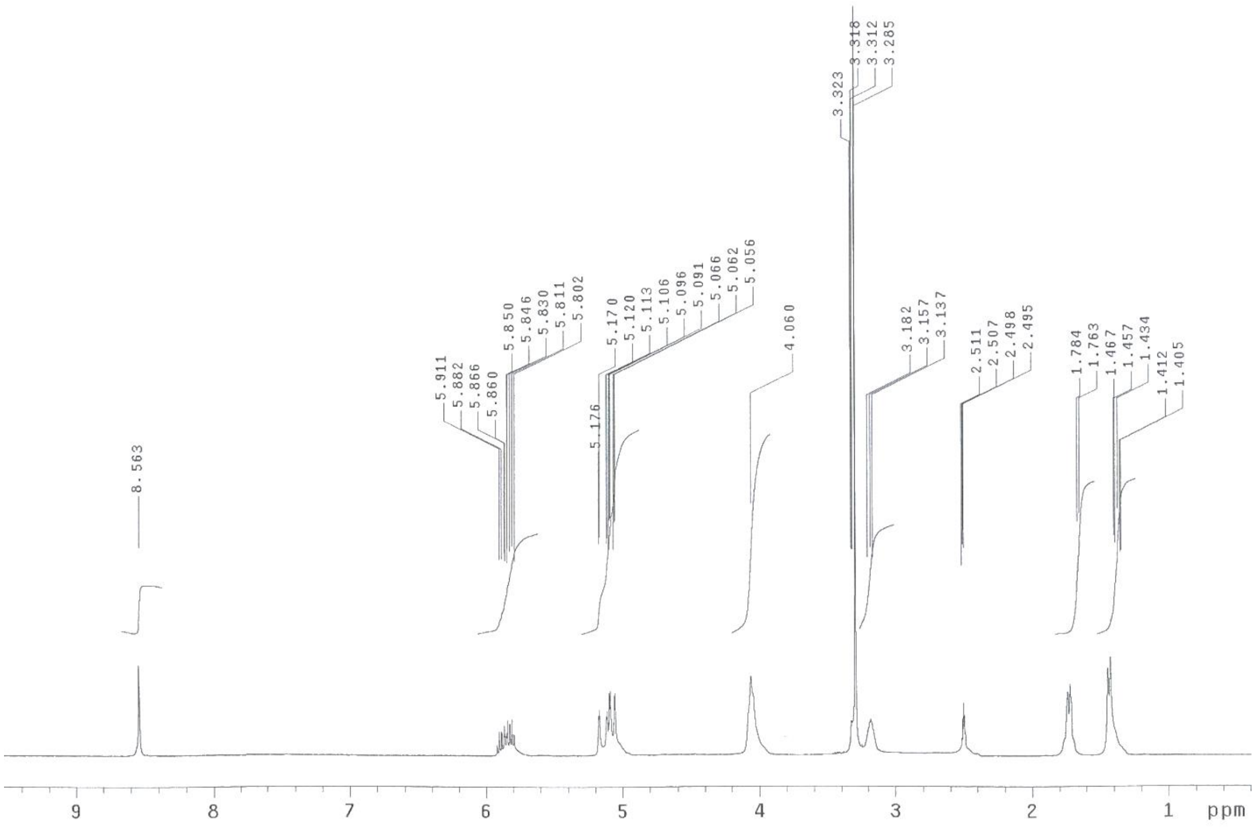
8.563
5.911
5.882
5.866
5.860
5.850
5.846
5.830
5.811
5.802
5.176
5.170
5.120
5.113
5.106
5.096
5.091
5.066
5.062
5.056
4.060
3.323
3.318
3.312
3.285
3.182
3.157
3.137
2.511
2.507
2.498
2.495
1.784
1.763
1.467
1.457
1.434
1.412
1.405
9
8
7
6
5
4
3
2
1
ppm

$^{1}$H NMR of **4b**

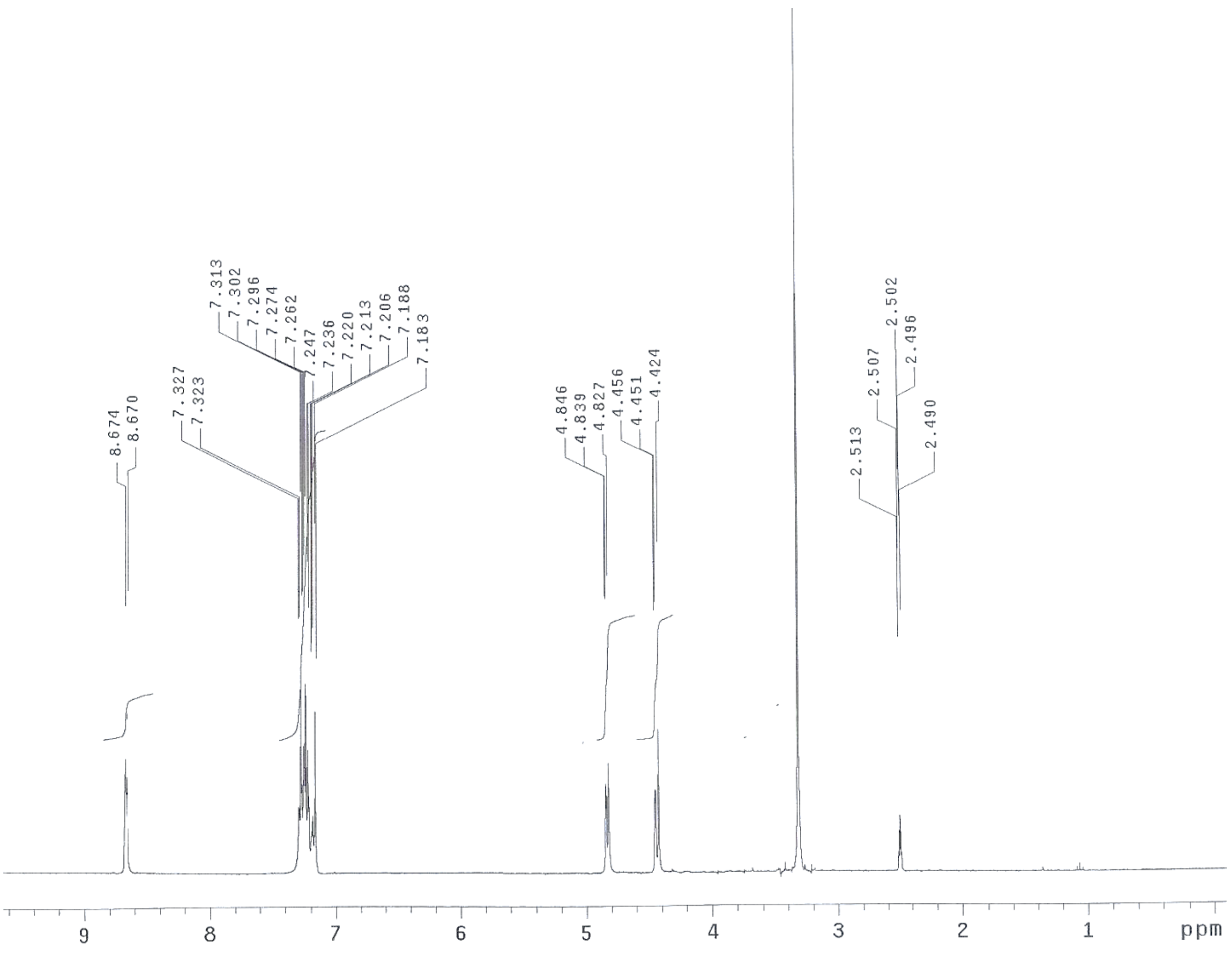


$^1$H NMR of **4c + 5a**

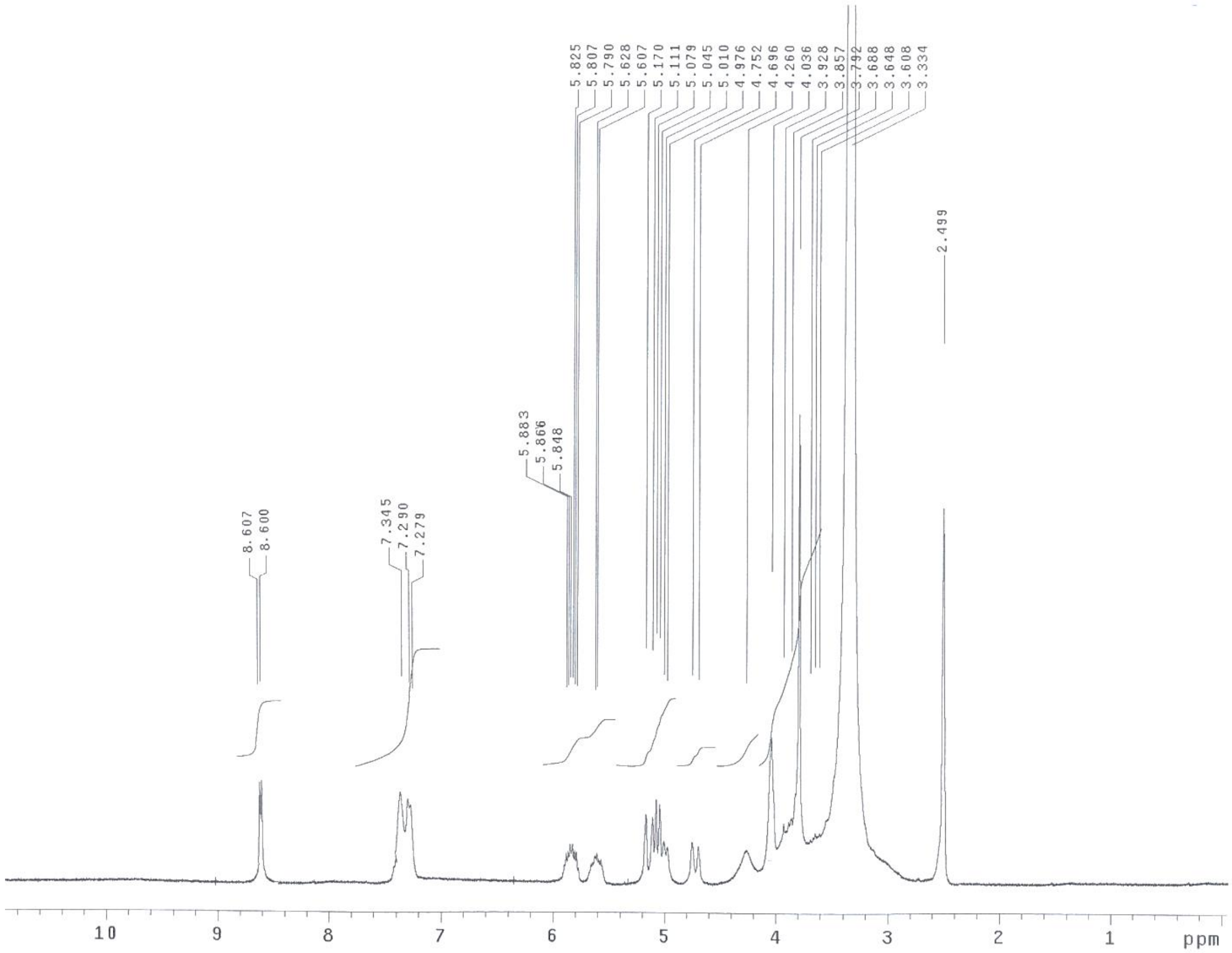


$^{1}$H NMR of **4d + 5b**

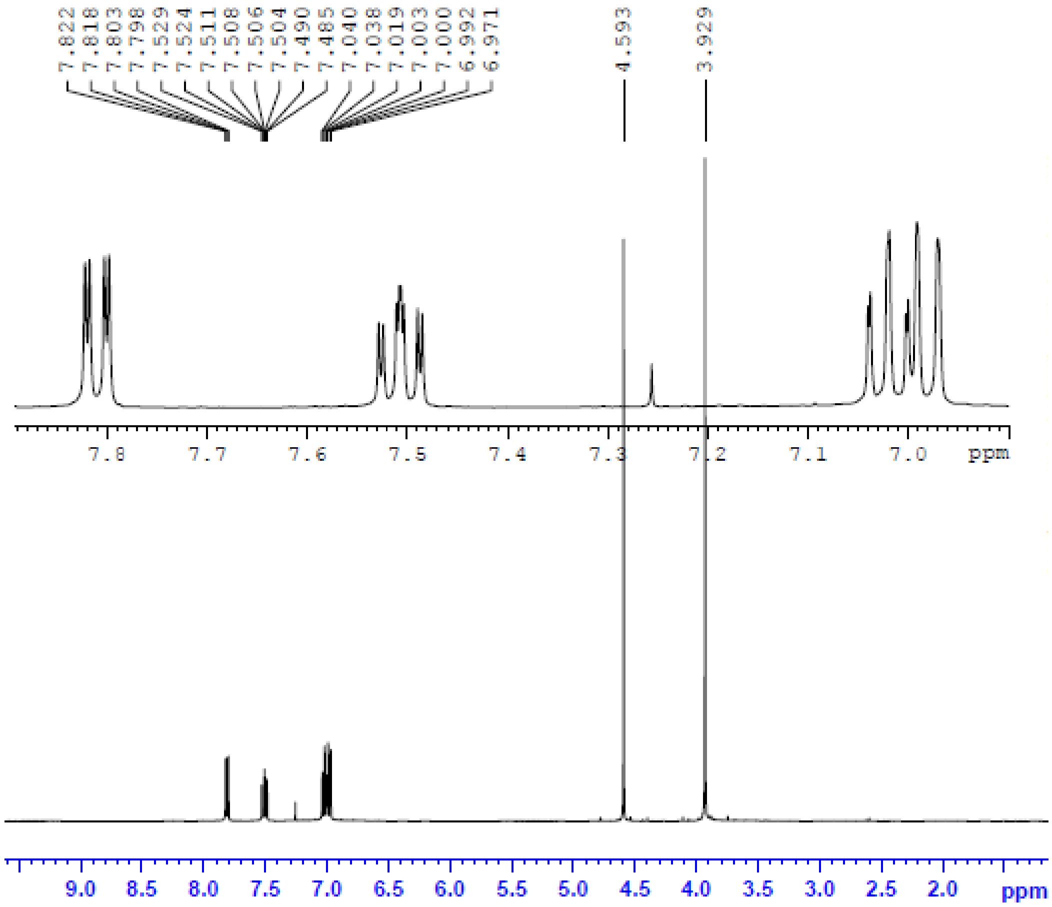
7.822
7.818
7.803
7.798
7.529
7.524
7.511
7.508
7.506
7.504
7.490
7.485
7.040
7.038
7.019
7.003
7.000
6.992
6.971
4.593
3.929
7.8
7.7
7.6
7.5
7.4
7.3
7.2
7.1
7.0
ppm
9.0
8.5
8.0
7.5
7.0
6.5
6.0
5.5
5.0
4.5
4.0
3.5
3.0
2.5
2.0
ppm

$^1$H NMR of **6**

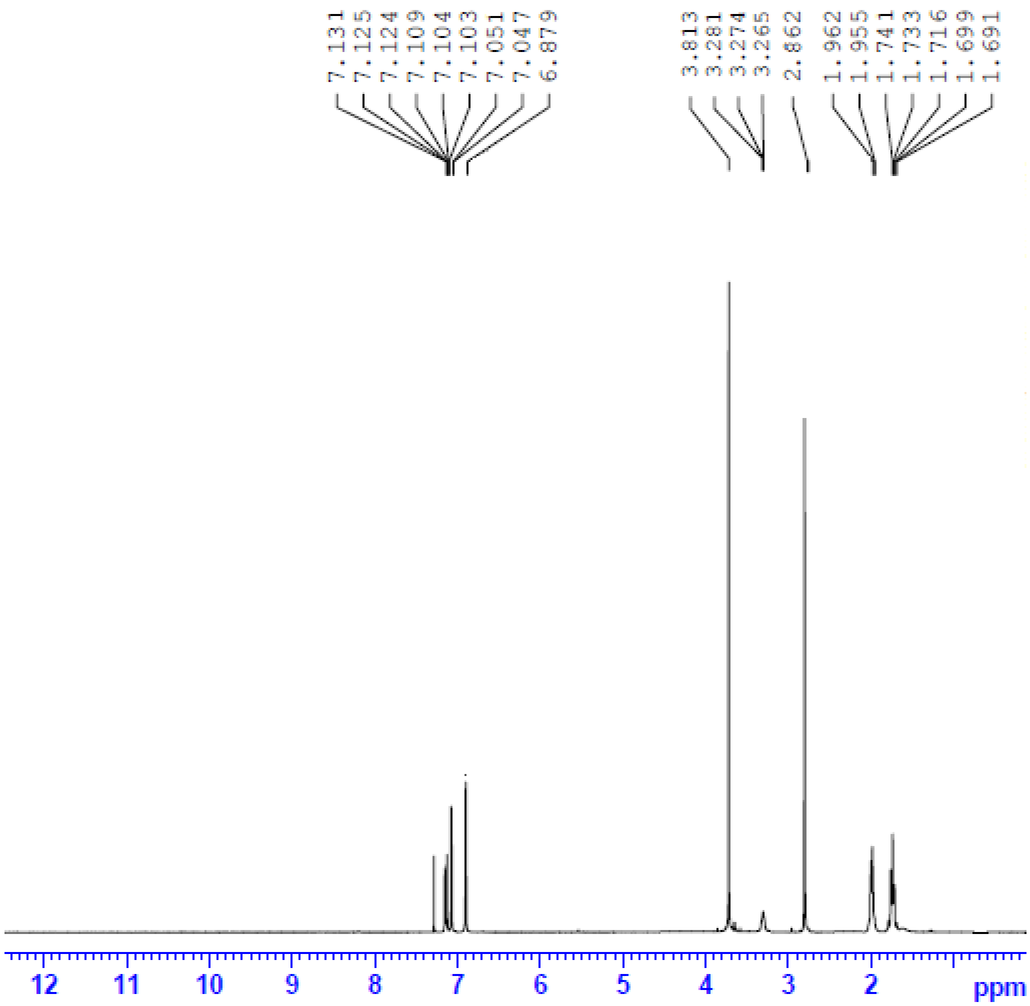
7.131
7.125
7.124
7.109
7.104
7.103
7.051
7.047
6.879
3.813
3.281
3.274
3.265
2.862
1.962
1.955
1.741
1.733
1.716
1.699
1.691
12
11
10
9
8
7
6
5
4
3
2
ppm

$^1$H NMR of **7a**

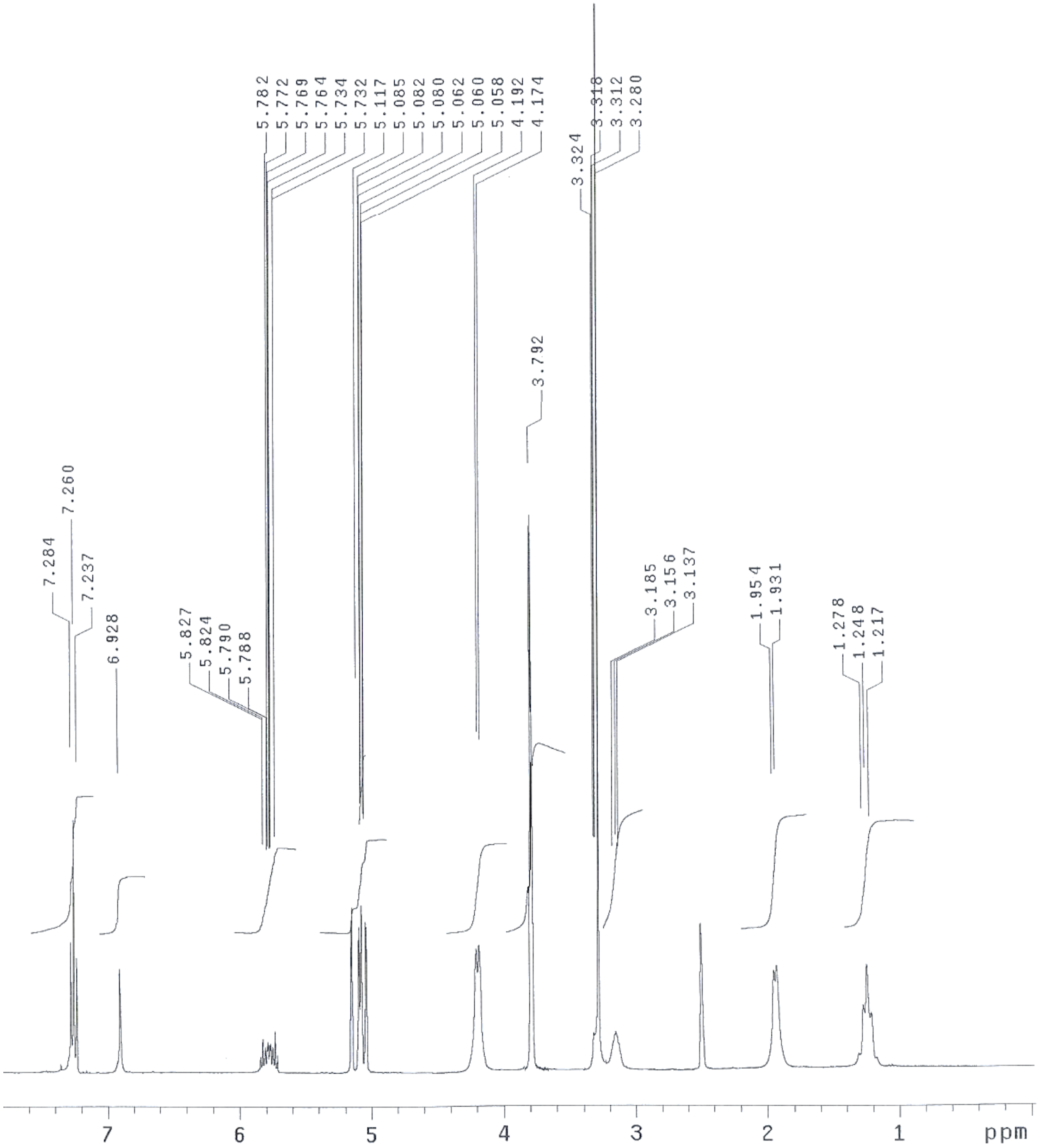
7.284
7.260
7.237
6.928
5.827
5.824
5.790
5.788
5.782
5.772
5.769
5.764
5.734
5.732
5.117
5.085
5.082
5.080
5.062
5.060
5.058
4.192
4.174
3.792
3.324
3.318
3.312
3.280
3.185
3.156
3.137
1.954
1.931
1.278
1.248
1.217
7
6
5
4
3
2
1
ppm

$^{1}$H NMR of **7b**

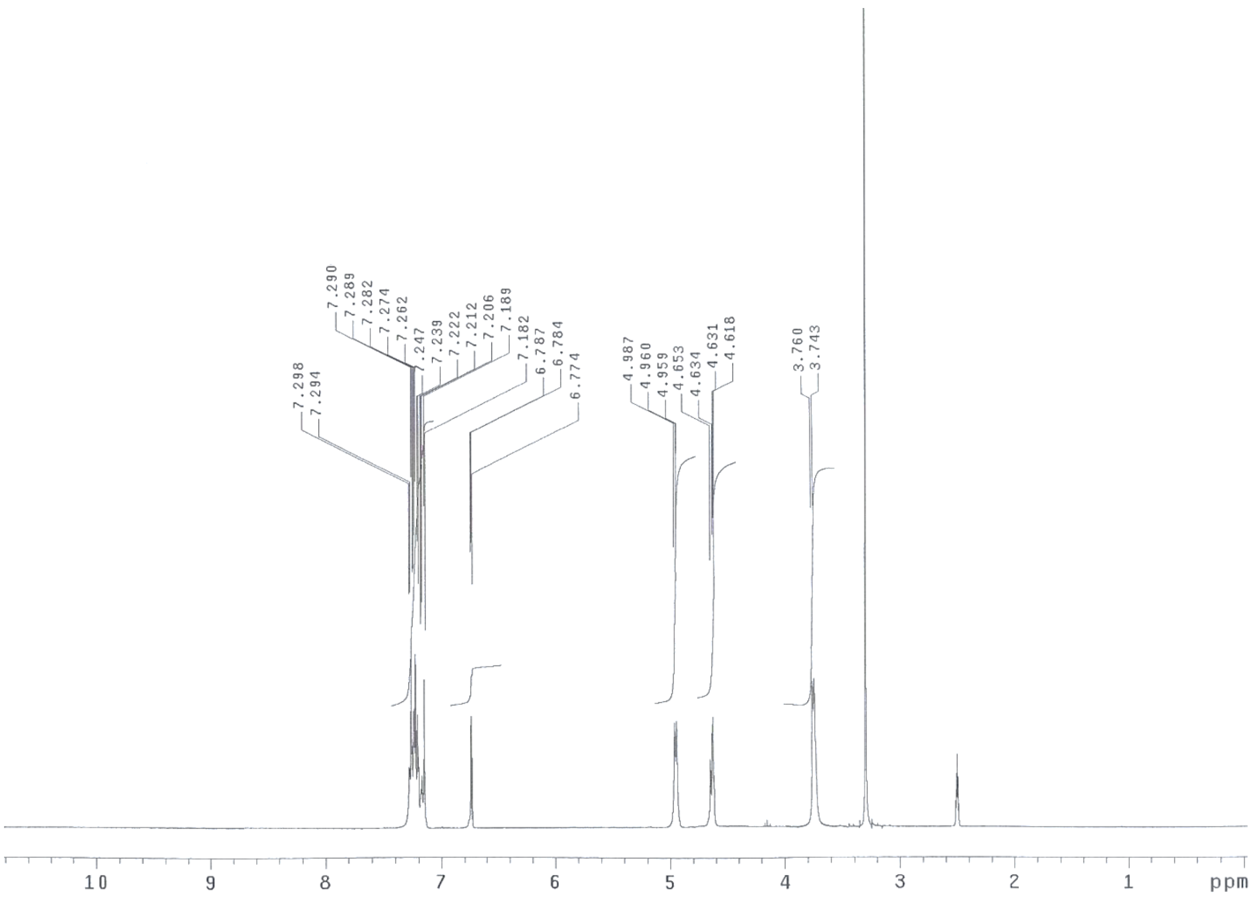

$^{1}$H NMR of **8a + 9a**

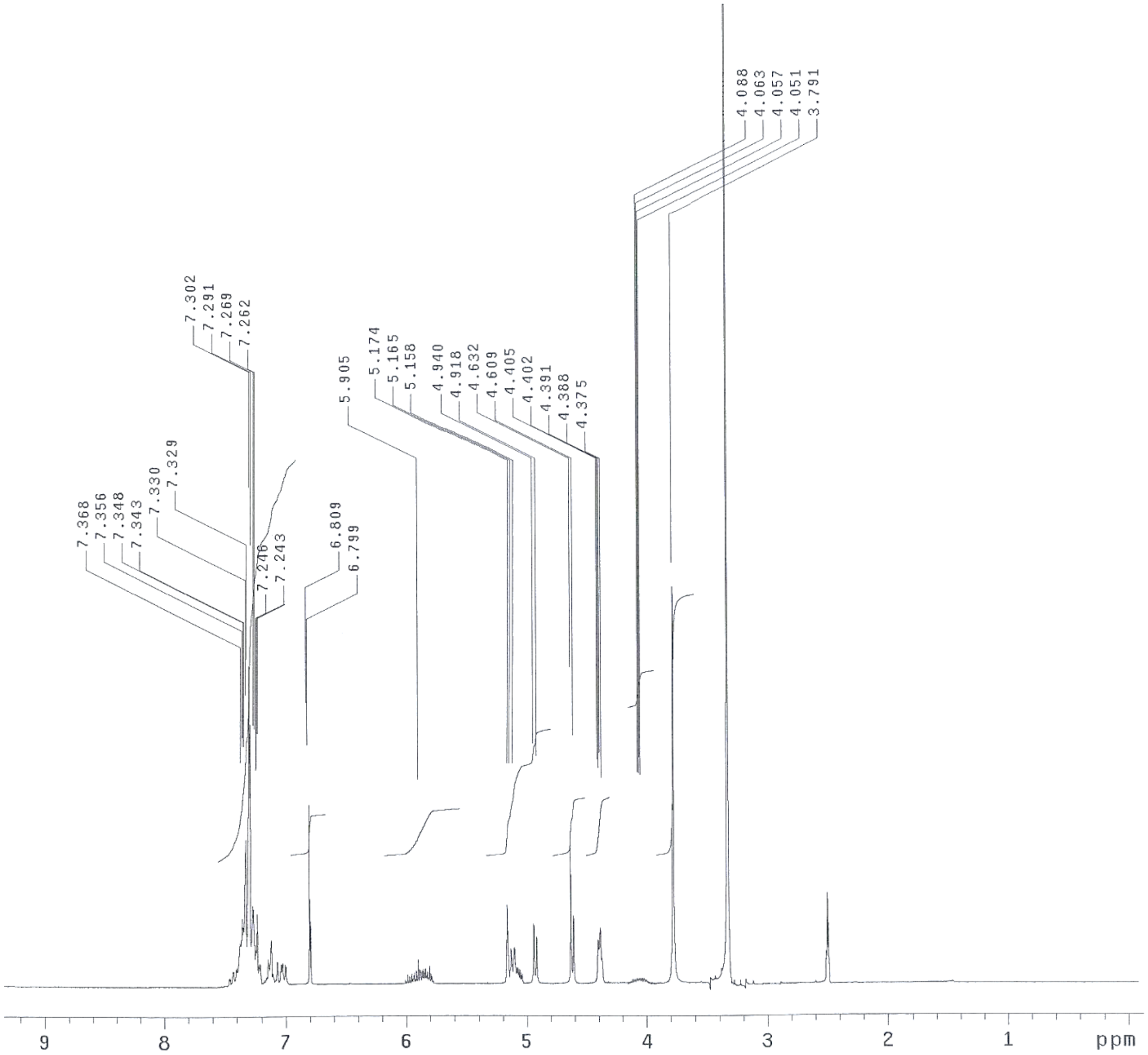


$^1$H NMR of **8b + 9b**

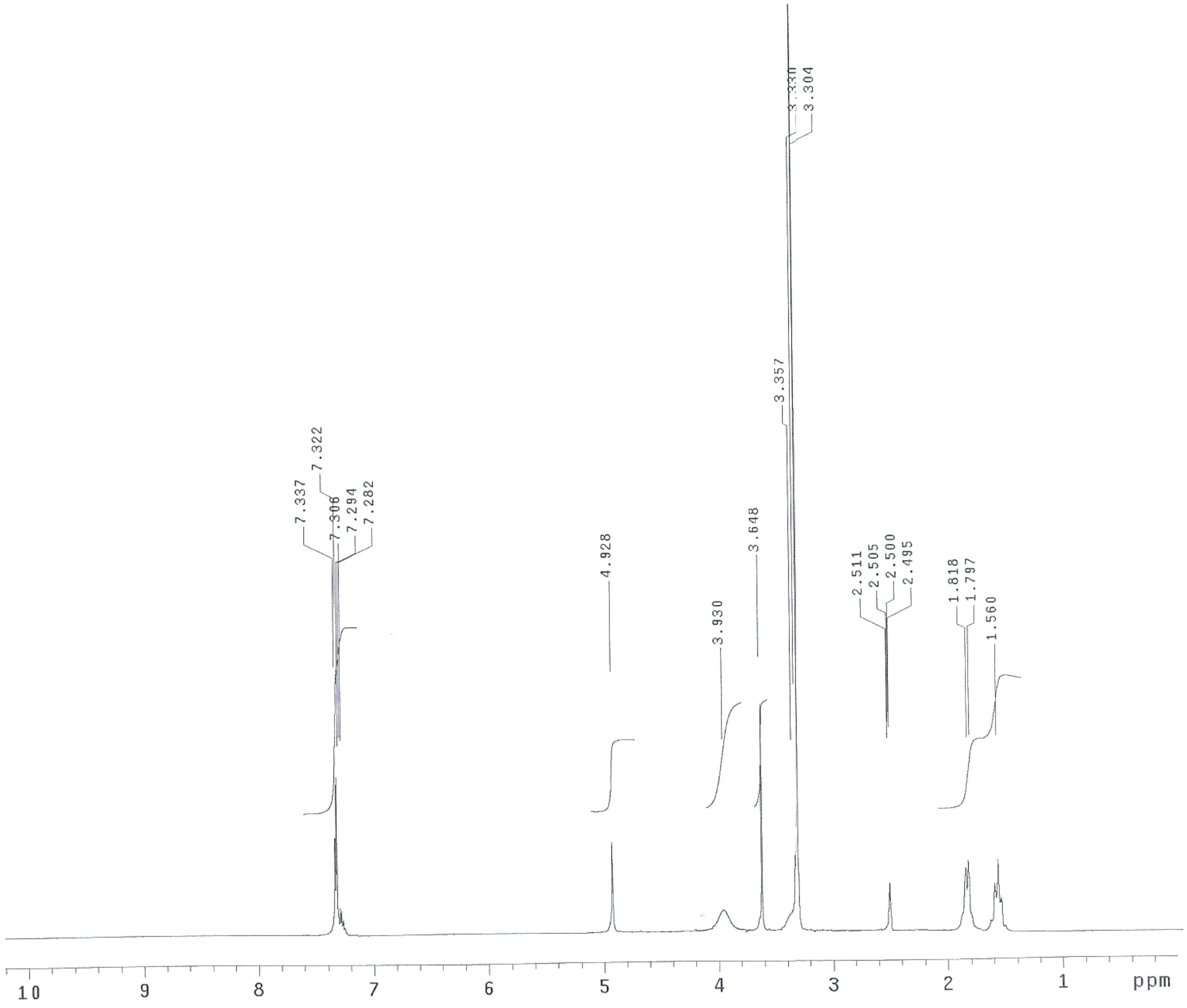
7.337
7.322
7.306
7.294
7.282
4.928
3.930
3.648
3.357
3.330
3.304
2.511
2.505
2.500
2.495
1.818
1.797
1.560
10
9
8
7
6
5
4
3
2
1
ppm

$^{1}$H NMR of **10a**

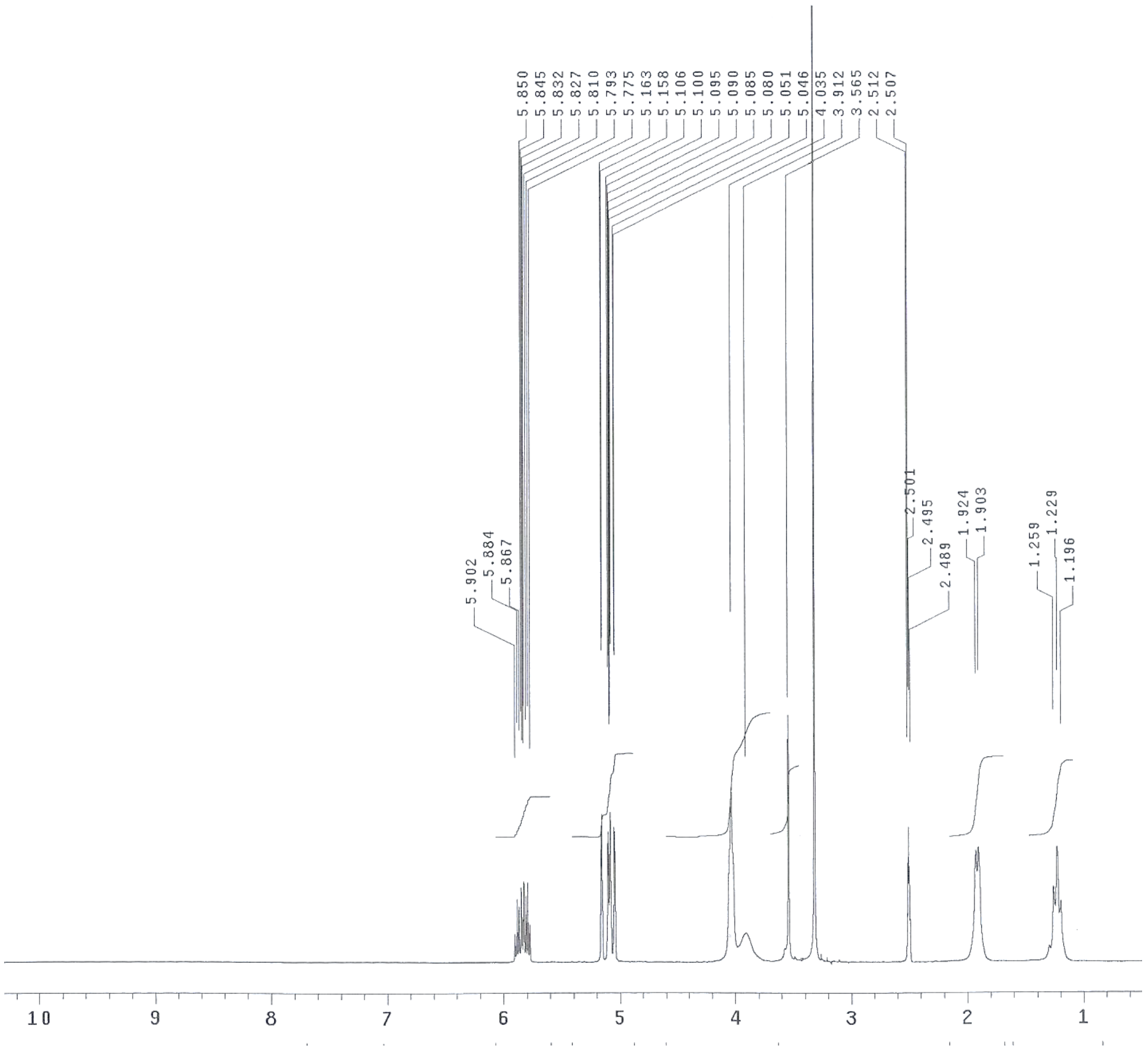
5.850
5.845
5.832
5.827
5.810
5.793
5.775
5.163
5.158
5.106
5.100
5.095
5.090
5.085
5.080
5.051
5.046
4.035
3.912
3.565
2.512
2.507
5.902
5.884
5.867
2.501
2.495
2.489
1.924
1.903
1.259
1.229
1.196
10
9
8
7
6
5
4
3
2
1

$^1$H NMR of **10b**

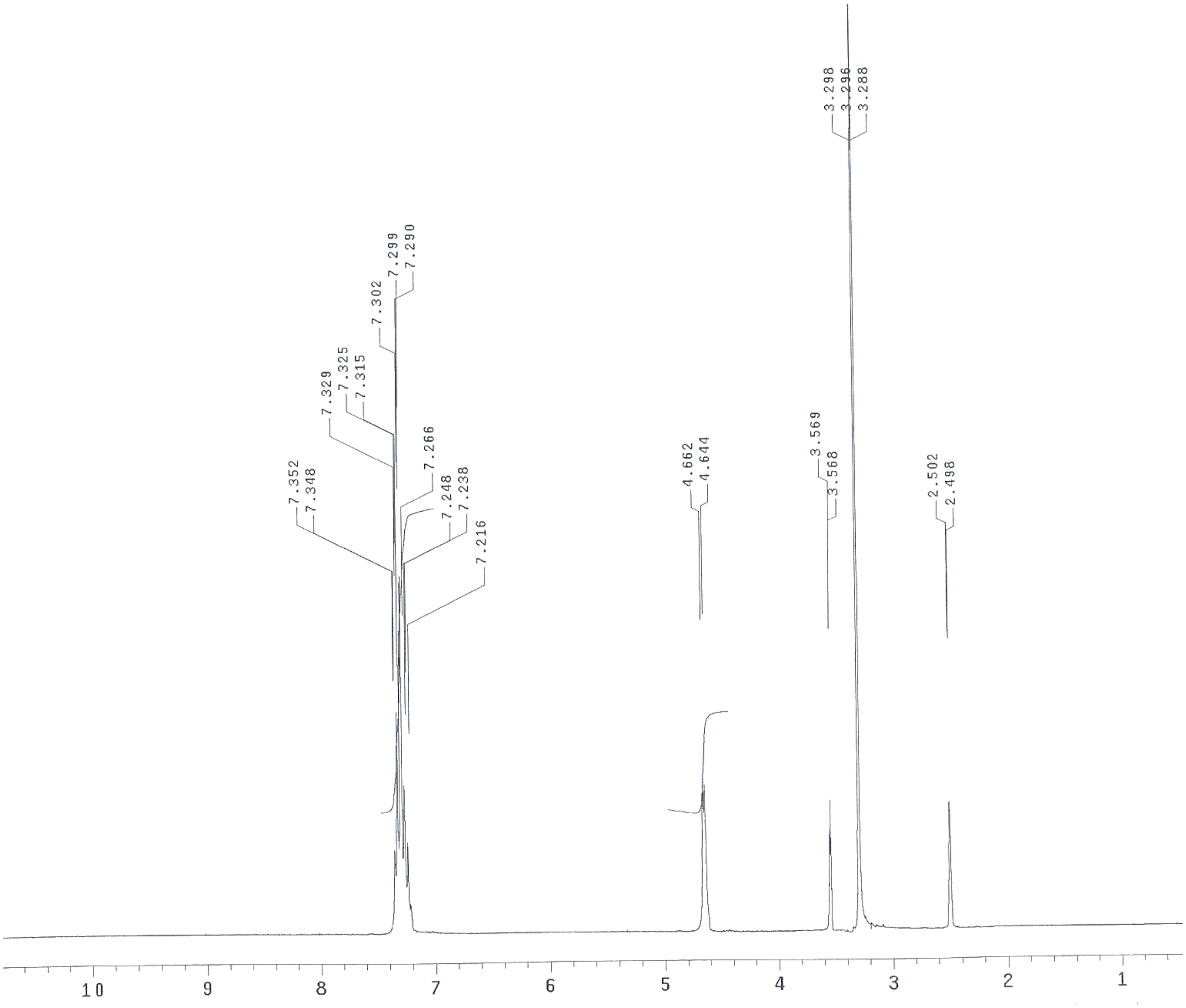
7.352
7.348
7.329
7.325
7.315
7.302
7.299
7.290
7.266
7.248
7.238
7.216
4.662
4.644
3.569
3.568
3.298
3.296
3.288
2.502
2.498
10
9
8
7
6
5
4
3
2
1

$^{1}$H NMR of **10c**

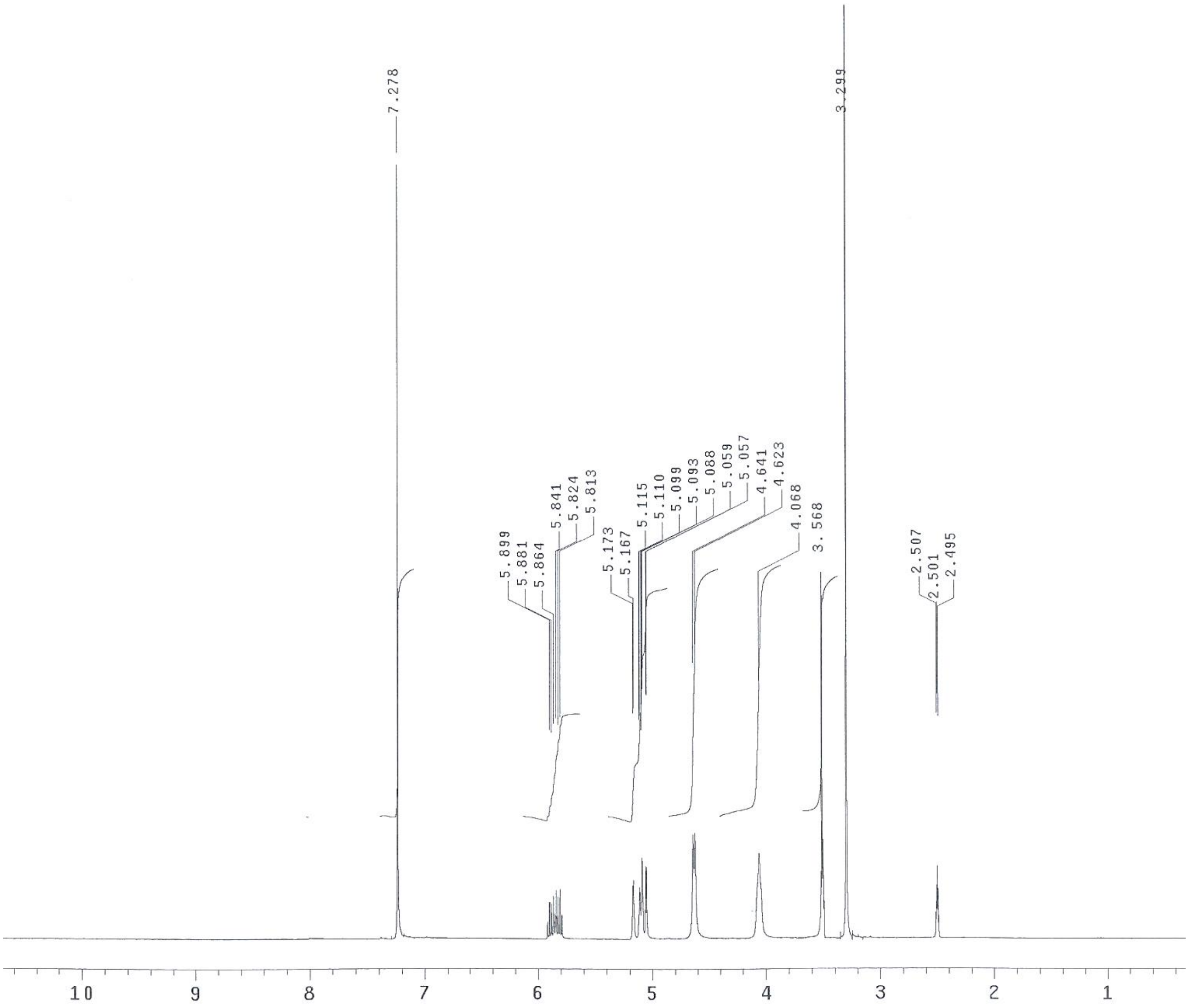
7.278
3.299
5.899
5.881
5.864
5.841
5.824
5.813
5.173
5.167
5.115
5.110
5.099
5.093
5.088
5.059
5.057
4.641
4.623
4.068
3.568
2.507
2.501
2.495
10
9
8
7
6
5
4
3
2
1

$^1$H NMR of **10d**

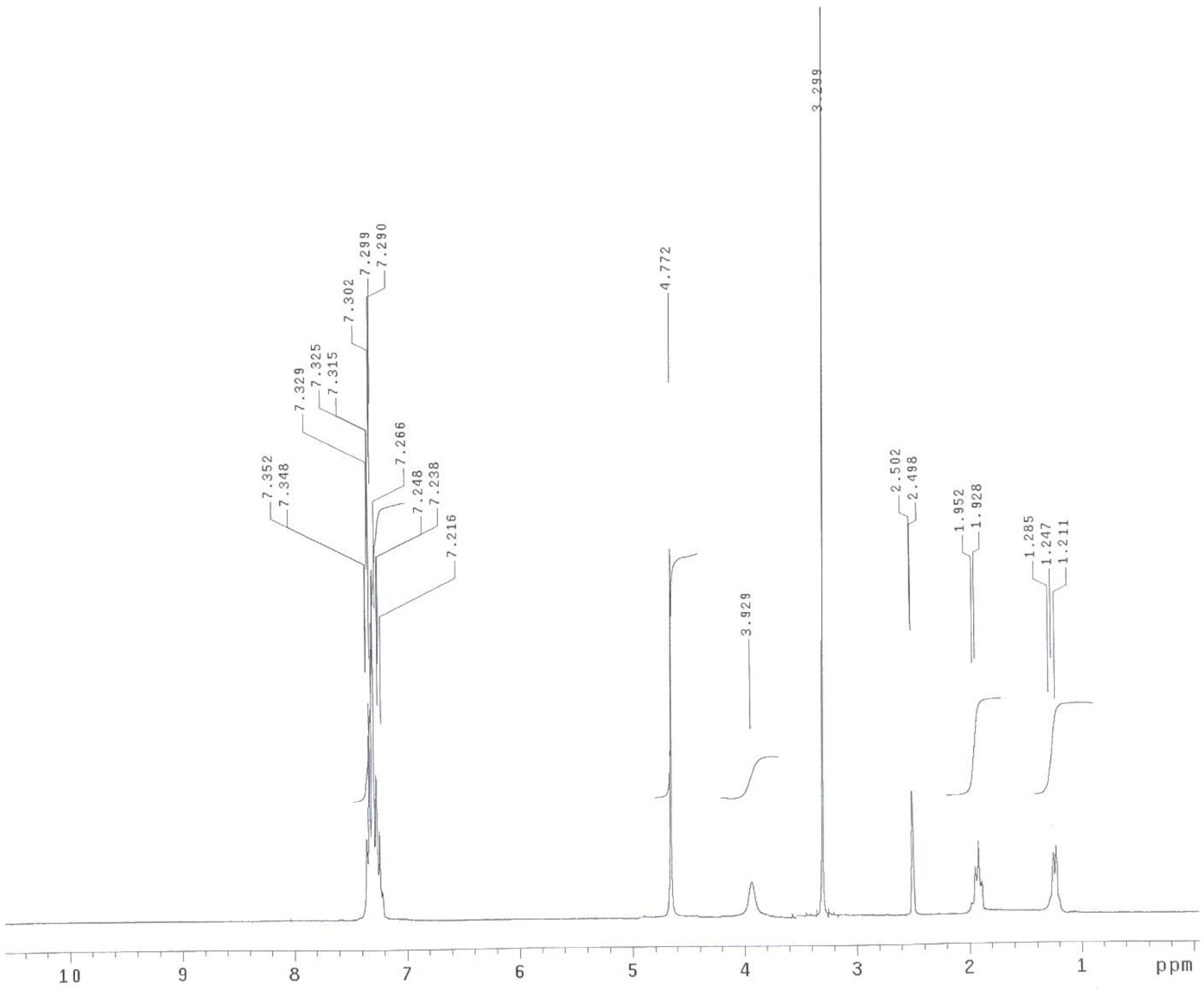
7.352
7.348
7.329
7.325
7.315
7.302
7.299
7.290
7.266
7.248
7.238
7.216
4.772
3.929
3.299
2.502
2.498
1.952
1.928
1.285
1.247
1.211
10
9
8
7
6
5
4
3
2
1
ppm

$^1$H NMR of **11a**

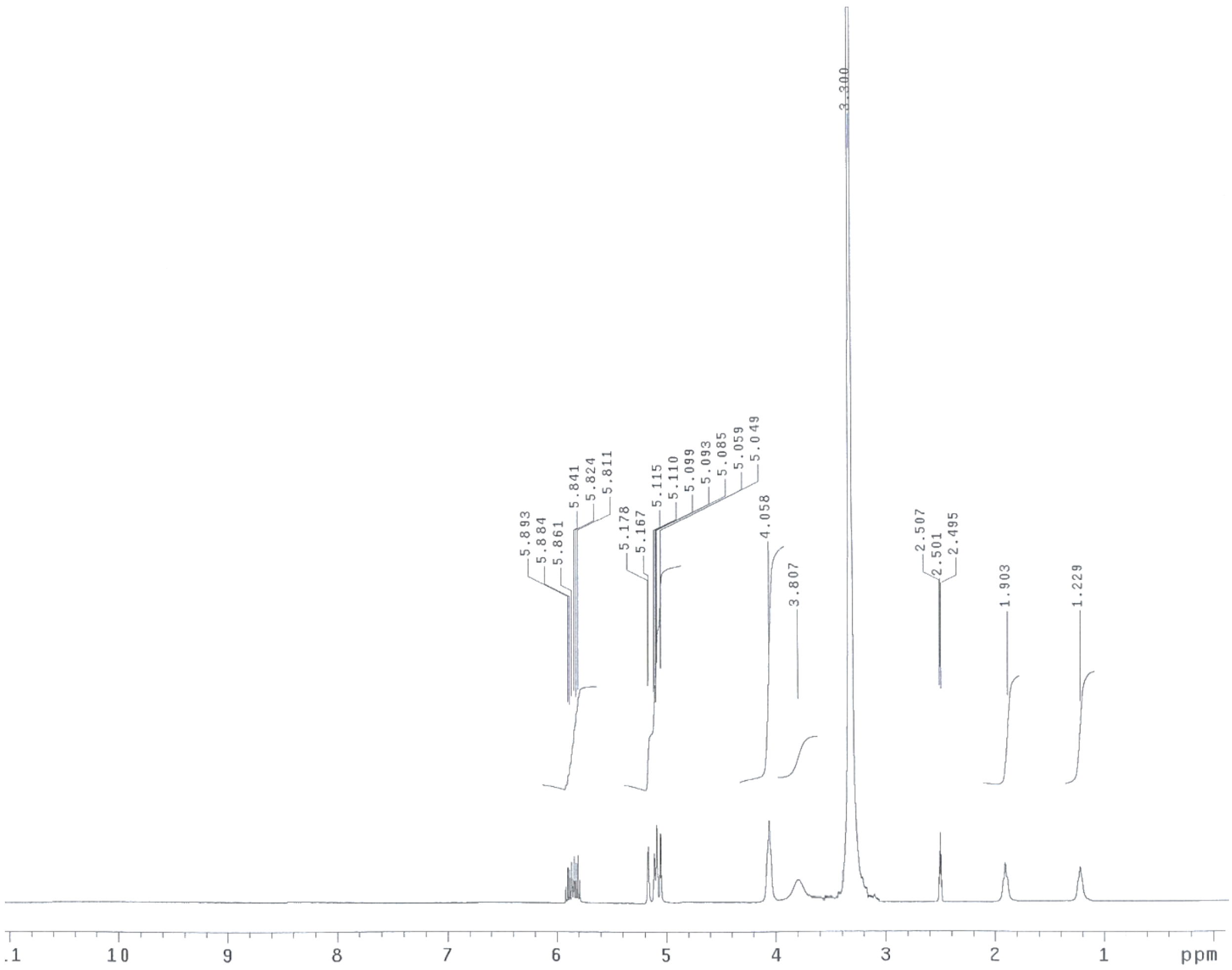
5.893
5.884
5.861
5.841
5.824
5.811
5.178
5.167
5.115
5.110
5.099
5.093
5.085
5.059
5.049
4.058
3.807
3.300
2.507
2.501
2.495
1.903
1.229
.1 10 9 8 7 6 5 4 3 2 1 ppm

$^{1}$H NMR of **11b**

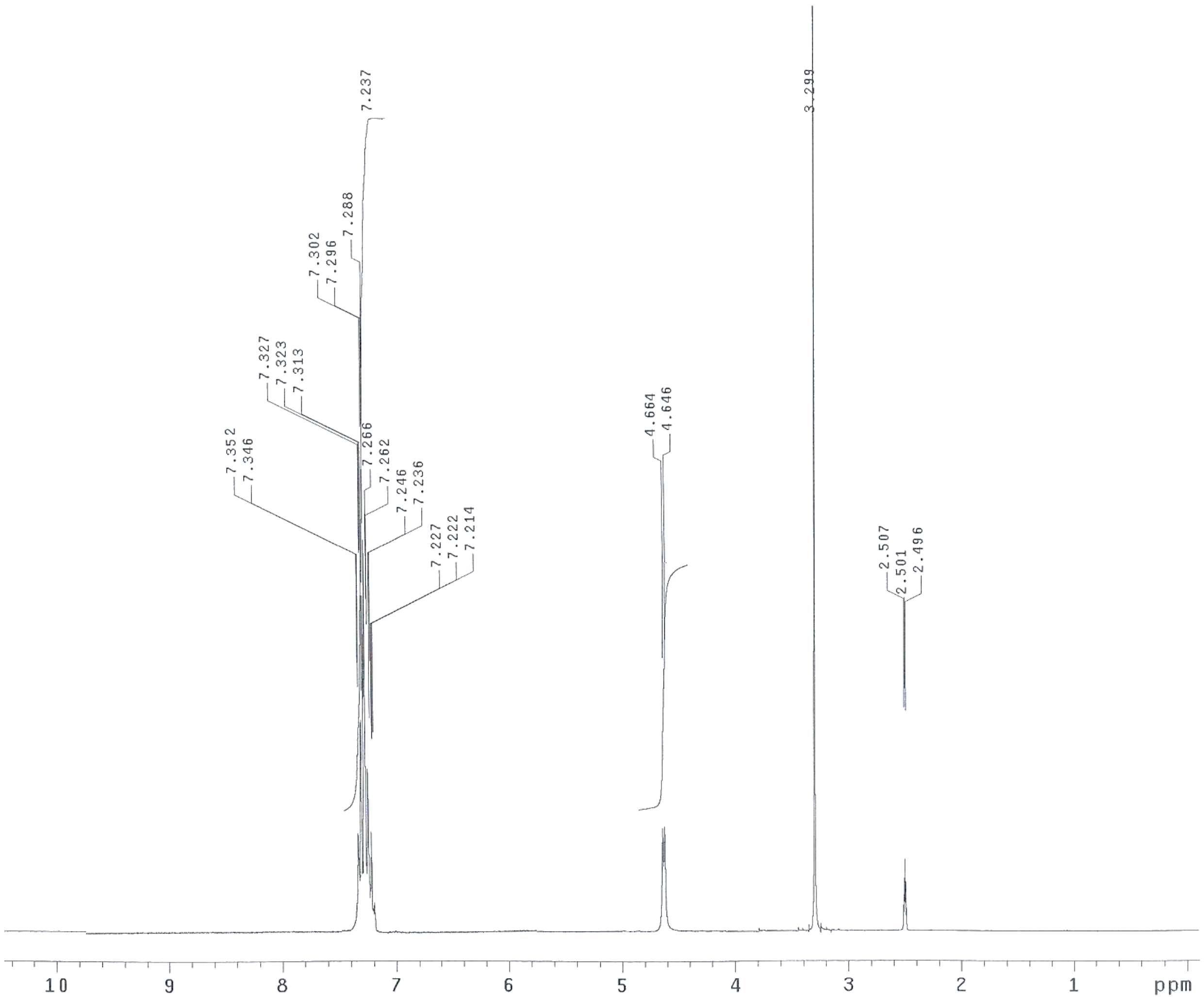
7.237
7.288
7.302
7.296
7.327
7.323
7.313
7.352
7.346
7.266
7.262
7.246
7.236
7.227
7.222
7.214
4.664
4.646
3.299
2.507
2.501
2.496
10
9
8
7
6
5
4
3
2
1
ppm

$^{1}H$ NMR of **11c**

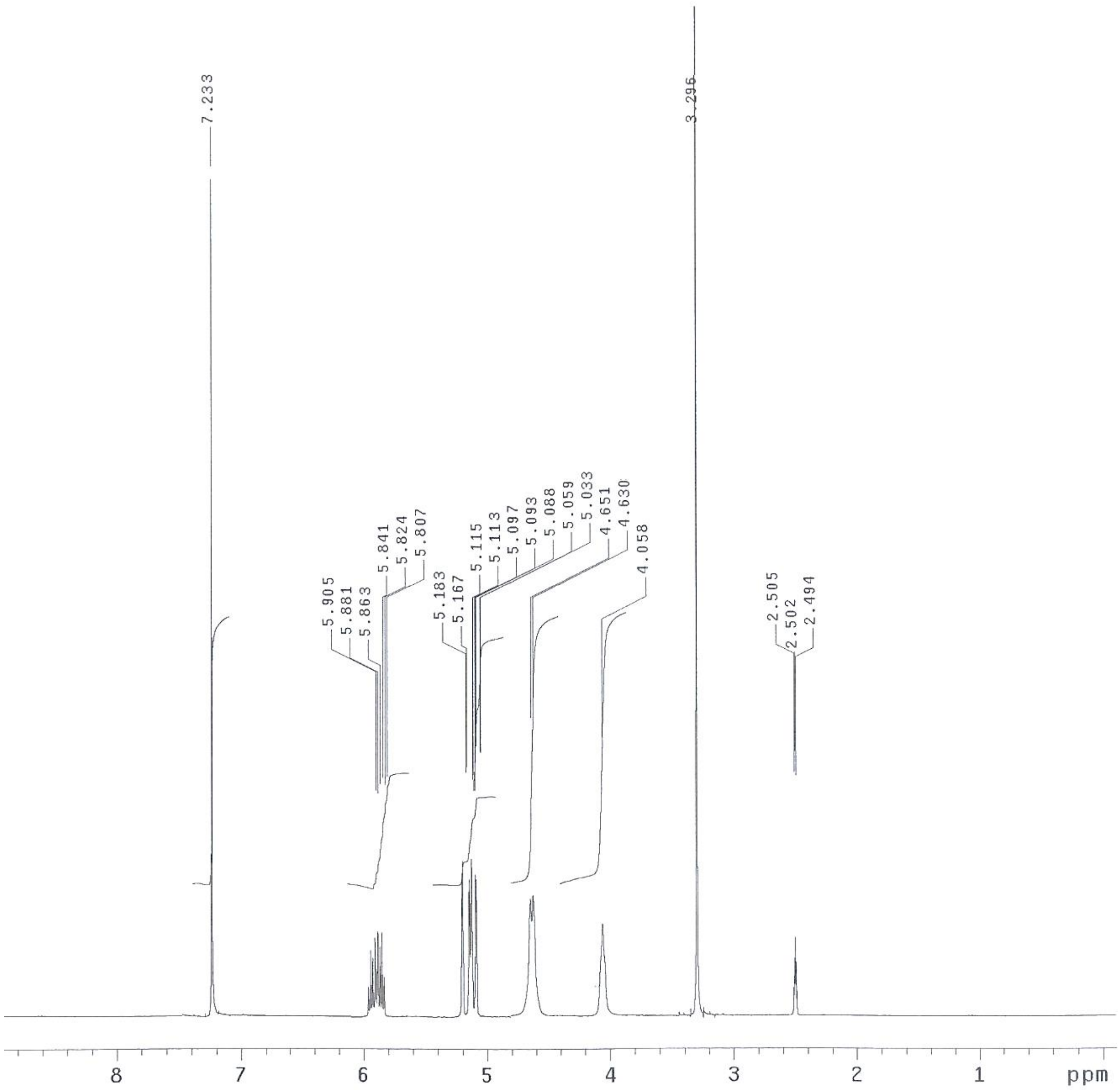
7.233
3.296
5.905
5.881
5.863
5.841
5.824
5.807
5.183
5.167
5.115
5.113
5.097
5.093
5.088
5.059
5.033
4.651
4.630
4.058
2.505
2.502
2.494
8
7
6
5
4
3
2
1
ppm

$^{1}H$ NMR of **11d**

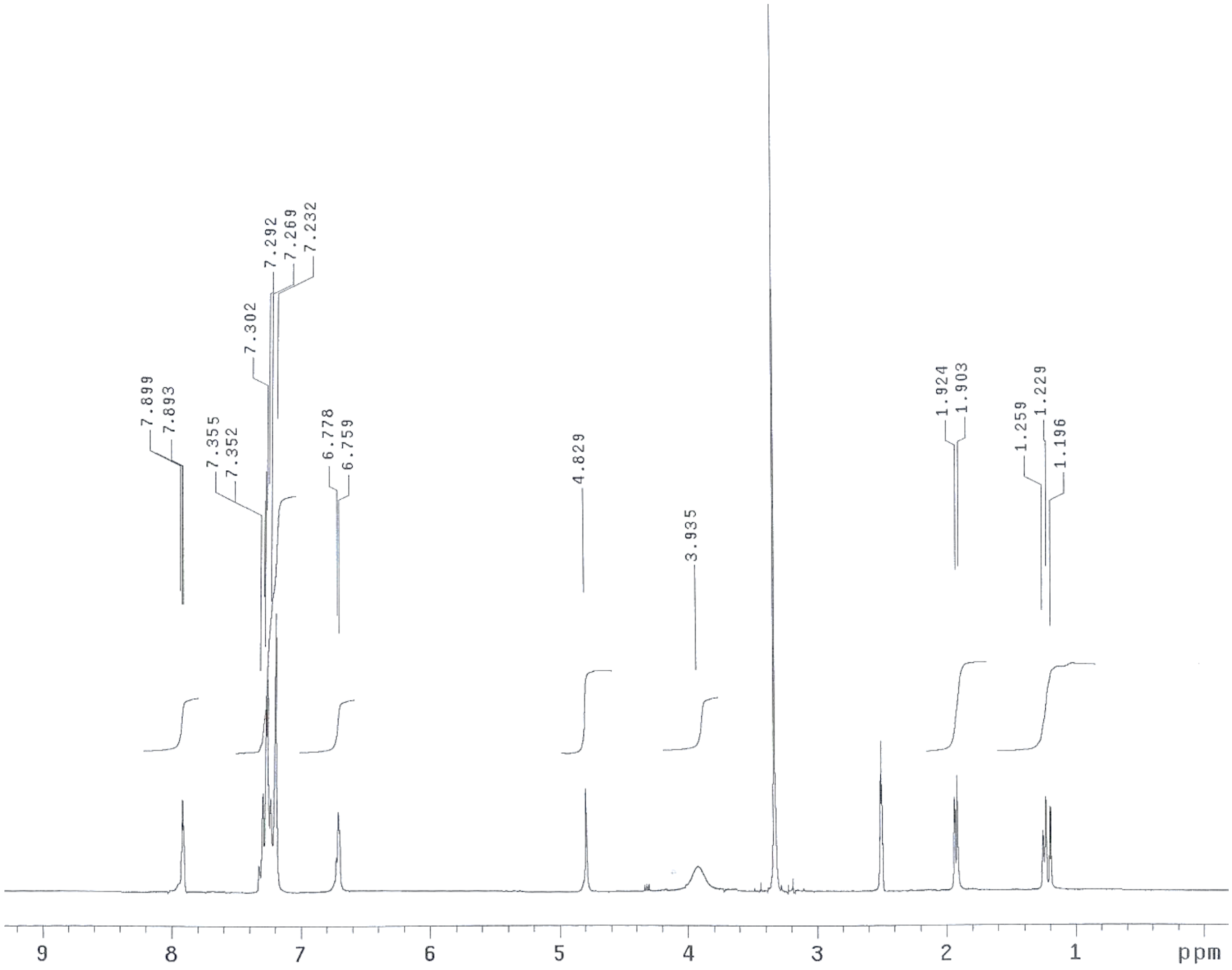
7.899
7.893
7.355
7.352
7.302
7.292
7.269
7.232
6.778
6.759
4.829
3.935
1.924
1.903
1.259
1.229
1.196
9
8
7
6
5
4
3
2
1
ppm

$^1$H NMR of **13a**

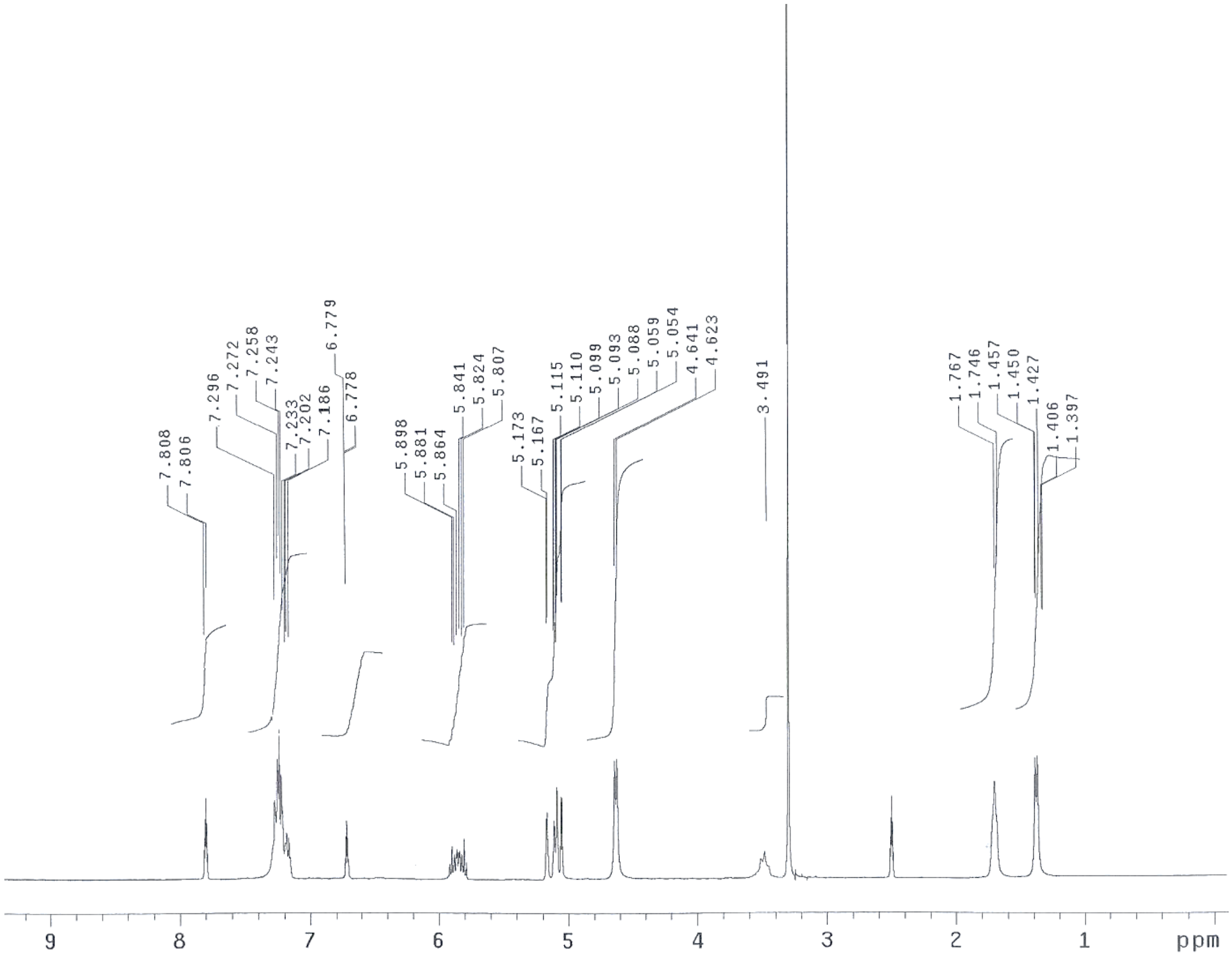

$^{1}$H NMR of **13b**

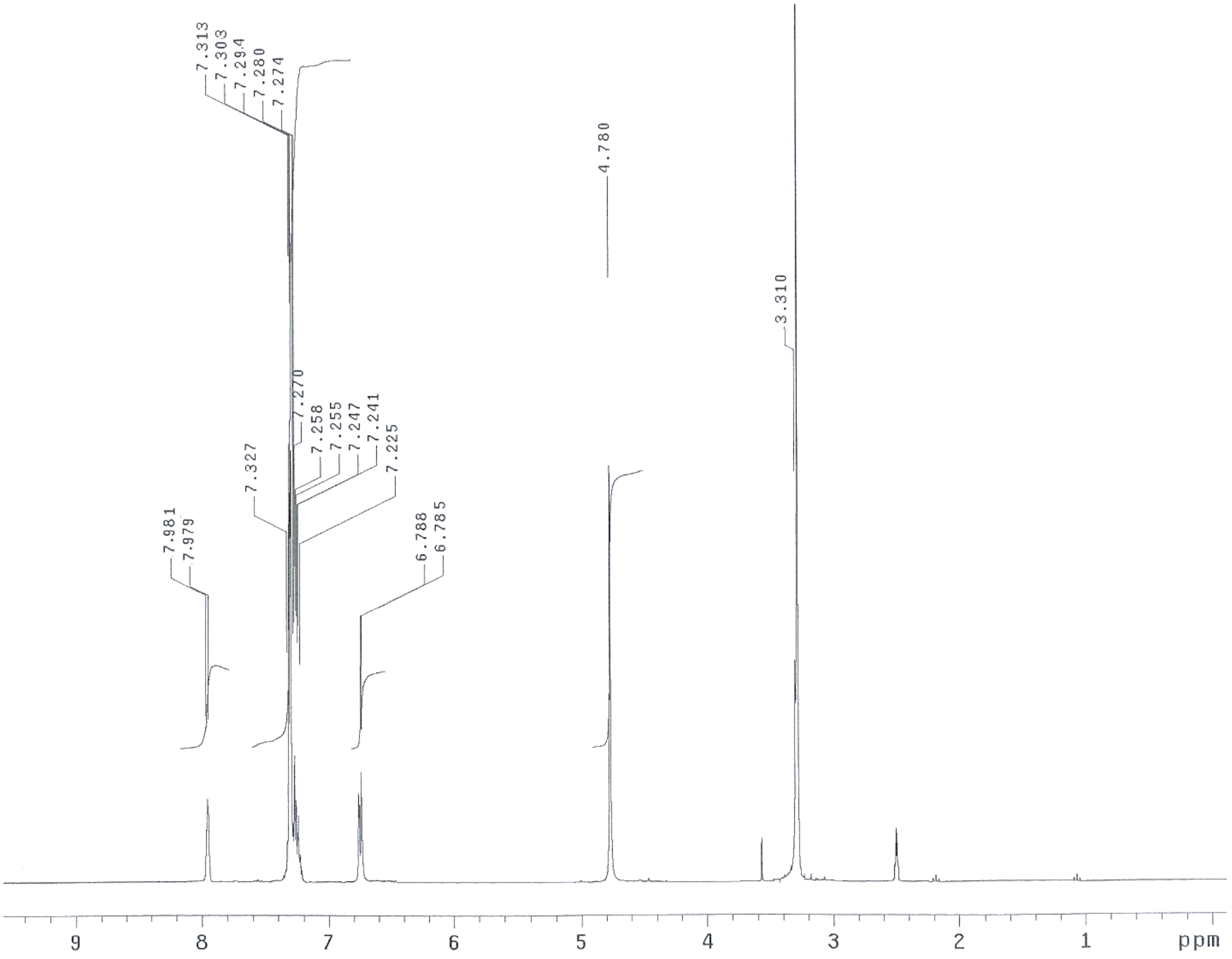
7.981
7.979
7.327
7.313
7.303
7.294
7.280
7.274
7.270
7.258
7.255
7.247
7.241
7.225
6.788
6.785
4.780
3.310
9
8
7
6
5
4
3
2
1
ppm

$^{1}$H NMR of **13c**

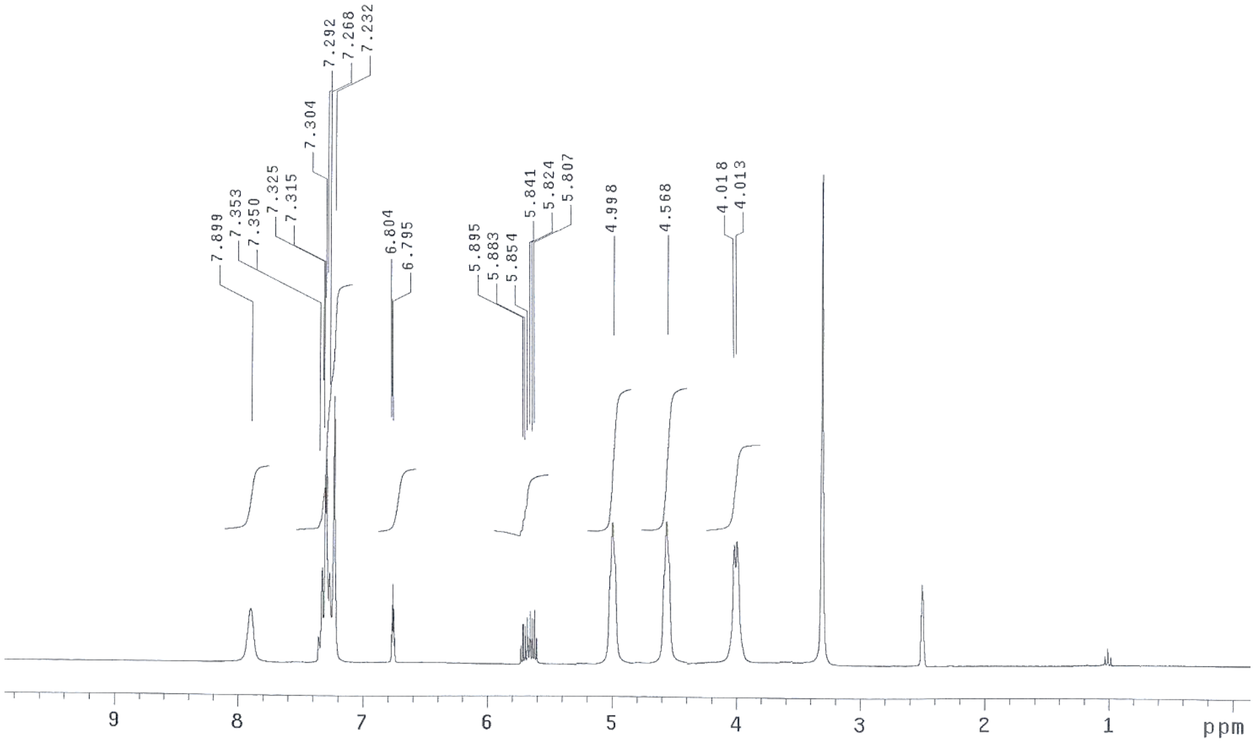
7.899
7.353
7.350
7.325
7.315
7.304
7.292
7.268
7.232
6.804
6.795
5.895
5.883
5.854
5.841
5.824
5.807
4.998
4.568
4.018
4.013
9
8
7
6
5
4
3
2
1
ppm

$^{1}$H NMR of **13d**

**Green Metrics Calculations**[1,2]

$$\textbf{\% Atomic Efficiency (AE)} = \frac{\text{Mol Wt. of desired product}}{\text{Mol Wt. of all reagents}} \times 100$$

$$\textbf{\% Carbon Efficiency (CE)} = \frac{\text{Mass of carbon in product}}{\text{Totall mass of carbon in the reactants}} \times 100$$

$$\textbf{Reaction Mass Efficiency (RME)} = \frac{\text{Mass of the isolated product}}{\text{Total mass of reactants used in the reaction}} \times 100$$

$$\textbf{E-Factor (EF)} = \frac{\text{Mass of the total waste}}{\text{Mass of the crude product}}$$

$$\textbf{Process Mass Intensity (PMI)} = \frac{\text{Total mass used in process}}{\text{Mass of product}}$$

## References


1. D. Curzons, D. J. C. Constable, D. N. Mortimer and V. L. Cunningham, *Green Chem.*, 2001, **3**, 1-6.
2. C. Jimenez-Gonzalez, D. J. C. Constable and C. S. Ponder, *Chem. Soc. Rev.*, 2012, **41**, 1485-1498.
